# DyslexiaNet: Examining the Viability and Efficacy of Eye Movement-Based Deep Learning for Dyslexia Detection

**DOI:** 10.3390/jemr18050056

**Published:** 2025-10-15

**Authors:** Ramis İleri, Çiğdem Gülüzar Altıntop, Fatma Latifoğlu, Esra Demirci

**Affiliations:** 1 Department of Biomedical Engineering, Faculty of Engineering, Erciyes University, Kayseri 38280, Türkiye; ramissileri@gmail.com; 2 Department of Child and Adolescent Psychiatry, Erciyes University Faculty of Medicine, Kayseri 38280, Türkiye; esrademirci@erciyes.edu.tr

**Keywords:** dyslexia, eye movements, electrooculogram (EOG), convolutional neural network, AlexNet, ResNet50, MobileNet, scalogram

## Abstract

Dyslexia is a neurodevelopmental disorder that impairs reading, affecting 5–17.5% of children and representing the most common learning disability. Individuals with dyslexia experience decoding, reading fluency, and comprehension difficulties, hindering vocabulary development and learning. Early and accurate identification is essential for targeted interventions. Traditional diagnostic methods rely on behavioral assessments and neuropsychological tests, which can be time-consuming and subjective. Recent studies suggest that physiological signals, such as electrooculography (EOG), can provide objective insights into reading-related cognitive and visual processes. Despite this potential, there is limited research on how typeface and font characteristics influence reading performance in dyslexic children using EOG measurements. To address this gap, we investigated the most suitable typefaces for Turkish-speaking children with dyslexia by analyzing EOG signals recorded during reading tasks. We developed a novel deep learning framework, DyslexiaNet, using scalogram images from horizontal and vertical EOG channels, and compared it with AlexNet, MobileNet, and ResNet. Reading performance indicators, including reading time, blink rate, regression rate, and EOG signal energy, were evaluated across multiple typefaces and font sizes. Results showed that typeface significantly affects reading efficiency in dyslexic children. The BonvenoCF font was associated with shorter reading times, fewer regressions, and lower cognitive load. DyslexiaNet achieved the highest classification accuracy (99.96% for horizontal channels) while requiring lower computational load than other networks. These findings demonstrate that EOG-based physiological measurements combined with deep learning offer a non-invasive, objective approach for dyslexia detection and personalized typeface selection. This method can provide practical guidance for designing educational materials and support clinicians in early diagnosis and individualized intervention strategies for children with dyslexia.

## 1. Introduction

Dyslexia is a neurodevelopmental learning disorder that impairs reading and is the most common type of learning disability, affecting approximately 5–17.5% of children and accounting for 70–80% of those with significant learning difficulties [1,2]. Despite adequate intelligence and educational opportunities, dyslexic individuals experience decoding, reading fluency, and comprehension difficulties, hindering vocabulary development and overall learning [3,4]. Early identification is crucial for initiating targeted interventions and supporting educational outcomes.

Traditional dyslexia diagnosis relies on behavioral assessments and standardized tests, including measures of phonological awareness, reading fluency, comprehension, and cognitive functions such as working memory and processing speed [5,6,7]. While effective, these methods are often time-consuming, subjective, and may under-identify bilingual or morphologically complex language speakers [8]. Teachers and families play a critical role in noticing early reading difficulties, emphasizing the need for continuous observation and comprehensive evaluation [9,10].

Recent research highlights physiological measurements as a promising complementary approach for dyslexia detection. Among these, Electrooculography (EOG) offers a non-invasive, cost-effective, and real-time method for tracking eye movements, including saccades, fixations, regressions, and blinks [11,12,13,14,15,16,17,18,19]. Unlike video-based systems, which require complex image processing algorithms [17], EOG provides a simpler and more accessible alternative, particularly in resource-constrained environments. Studies consistently show dyslexic readers exhibit longer fixations, more regressions, and shorter saccades than typical readers [20,21]. For example, Bachmann et al. [22] had typically developing controls and dyslexic children read Italian texts in two different fonts. They examined them in terms of fluent reading (number of syllables per second) and revealed whether there was a statistical difference in mean and standard deviation values. Rello et al. [23] used a statistical model to predict readers (Spanish readers) with and without dyslexia (from 11 to 54 years old) using eye-tracking measures, achieving 80.18% accuracy with features such as reading time, mean of fixations, and participant age. Furthermore, typographic features—such as font type, spacing, and character shape—can significantly influence reading efficiency, with specialized fonts like Dyslexie or OpenDyslexic designed to reduce common reading errors in dyslexic individuals [24,25,26]. These findings underscore the importance of typographic choices in educational and digital environments, suggesting that font design can serve as a low-cost, scalable intervention to support dyslexic readers.

Turkish varies from many other languages in two major ways. The first is that it has a completely transparent orthography. In other words, it is a language that is read exactly as it is written, with each letter of the alphabet representing a sound. The second is that it is an agglutinative language, which means it has a complex morphological structure [27]. According to [28], the integration of orthographic and morphological structures in Turkish does not provide readers with the predicted advantage of clear orthography. Unlike English, Turkish exhibits a near-perfect phoneme-to-grapheme correspondence, which minimizes decoding errors and allows researchers to isolate cognitive deficits unrelated to orthographic ambiguity [29]. Despite this regularity, dyslexic readers of Turkish still demonstrate characteristic reading impairments, including increased fixation durations, frequent regressions, and letter position errors [29,30,31]. The morphological richness of Turkish, with its extensive use of suffixes and compound word formation, further enables the examination of how dyslexia affects morphological parsing and lexical access. These features make Turkish a valuable language for cross-linguistic dyslexia research, offering insights into the interplay between language structure and reading disorders [29,30,31]. Comparative research on the influence of Turkish on dyslexia is considered necessary to adequately disclose the challenges that Turkish children face [32,33].

The diagnosis of dyslexia involves identifying and assessing reading-related learning impairments [34]. Traditional diagnostic and screening methods require professionals to conduct lengthy, face-to-face evaluations that measure reading and writing performance, including reading rate (words per minute), reading errors, writing mistakes, comprehension, pseudoword reading, and reading fluency [35]. In recent years, machine learning techniques have been increasingly applied to dyslexia detection, utilizing algorithms such as Support Vector Machines (SVM), Logistic Regression (LR), Artificial Neural Networks (ANN), Random Forests (RF), and k-Nearest Neighbors (KNN) [36]. These models are trained on features extracted from diverse signal modalities, including electroencephalography (EEG), electrooculography (EOG), and eye-tracking data.

Convolutional Neural Networks (CNNs) have demonstrated notable success in dyslexia detection, commonly employing data such as EEG, magnetic resonance imaging (MRI), handwriting samples, and eye movement recordings [37,38]. Studies have incorporated reading tasks in multiple languages, such as English, Spanish, and Swedish, to enhance generalizability [39,40,41]. For instance, Sait et al. [42] proposed a lightweight and interpretable deep learning framework integrating cross-modality data—specifically MRI, EEG, and handwriting images—for dyslexia detection, achieving 99.8% accuracy across five publicly available datasets and outperforming conventional CNNs and vision transformer architectures.

Building on these findings, the present study investigated whether dyslexia could be objectively detected by classifying EOG signals using deep neural networks. Children diagnosed with dyslexia were exposed to reading tasks with texts presented in various typefaces and font sizes through a controlled reading test system. This unique system is prepared in Turkish and allows the assessment of multiple typefaces and fonts, providing a novel approach to combining physiological measurements with typographic analysis for dyslexia detection and educational support.

**Figure 1:**
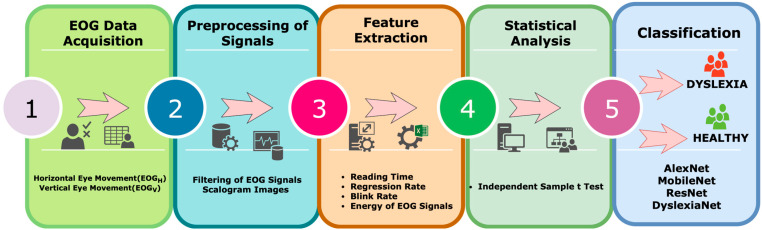
Overview of the proposed methodology.

To the best of our knowledge, no study has yet explored the combination of EOG-based physiological measurements with typeface analysis in children with dyslexia. This study addresses this gap by developing a reading task-based test system and recording EOG signals while children read texts in multiple typefaces and font sizes. Signal processing techniques and a novel deep learning approach based on EOG scalograms, DyslexiaNet, were applied to classify dyslexic and typical readers and determine optimal typefaces for reading efficiency. The findings aim to provide objective data for personalized educational support and assist clinicians in the early diagnosis of dyslexia. The flow chart of this study is given in Figure 1.

The paper is organized as follows: Section 2 presents the detailed information and methodology of the proposed approach; Section 3 shows experimental results and the model performance; Section 4 proceeds with discussions; and Section 5 is the final section, concluding this paper.

## 2. Materials and Methods

### 2.1. Data Acquisition

**Figure 2:**
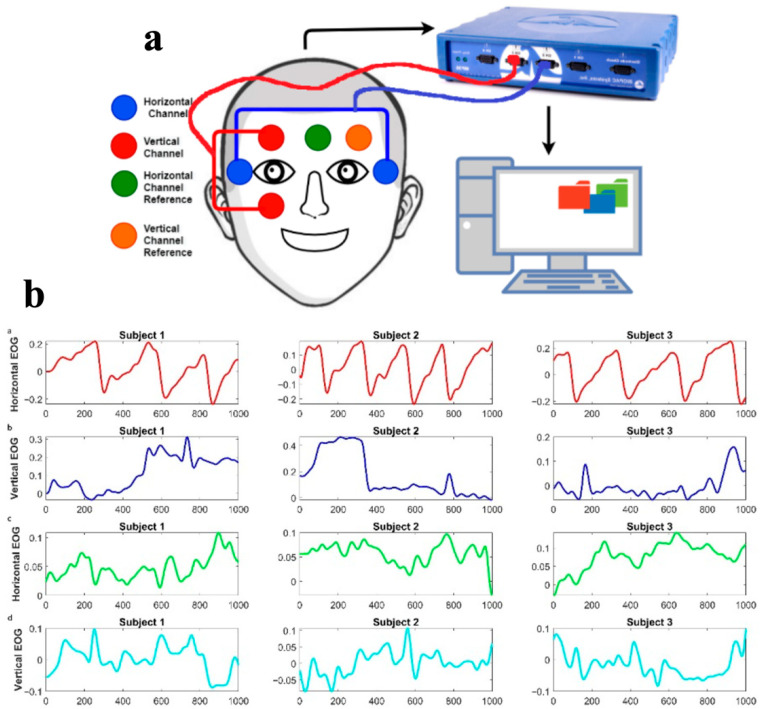
(**a**) The EOG electrode placement, (**b**) EOG signals of three random subjects while reading text: (**b.a**) horizontal EOG from TDC subject (**b.b**) vertical EOG from TDC subject, (**b.c**) horizontal EOG from dyslexic subject (**b.d**) vertical EOG from dyslexic subject.

In this paper, a reading task-based testing system was designed for data recording. Figure 2a illustrates the data acquisition system and electrode placement. EOG signals were obtained at a sampling frequency of 100 Hz. For data pre-processing, the raw EOG signals were filtered using a 4th-order Butterworth band-pass filter for noise removal. The frequency ranges from 0.1 to 10 Hz. Figure 2b shows the sample EOG signals from three random subjects from the second-, third-, and fourth-grade students.

### 2.2. Participants

Twenty-three children with dyslexia, aged 8–10 (13 females and 10 males), and 13 age–sex matched typically developing controls (TDC) were included in the study. All subjects were primary school second (7–8 years old), third (8–9 years old), and fourth (9–10 years old) grade students. TDC received a typical clinical evaluation that included assessments of her neurological, endocrine, and mental conditions. The study excluded children with dyslexia who also had other psychiatric illnesses, epilepsy, cerebral palsy, developmental delay, or other abnormalities of the central nervous system. The Diagnostic and Statistical Manual of Mental Disorders, Fifth Edition (DSM-5) criteria, as well as the specific learning disability (SLD) battery, which includes subtests that evaluate literacy and basic arithmetic skills, as well as tests that assess disorders or problems in visual perception, ranking and sequencing skills, the hand-eye-ear test of the head, lateralization, an assessment of lateralization, and an assessment of lateralization, were all used to make the diagnosis of dyslexia by child and adolescent psychiatrist. Ethical approval for this study was obtained from the ethics committee of Erciyes University, Kayseri (approval number: 2018/565). Verbal consent was obtained from the children participating in the study, and written consent was obtained from their parents. Supplementary Figure S1 and Table S2 show the subject’s characteristics.

### 2.3. Experimental Setup

Detailed information can be found about the experimental setup and the EOG Data acquisition system in [38]. A total of 28 distinct reading tasks were prepared for use in this study. These texts were designed in the participants’ native language (Turkish) and varied systematically in typeface and font size. The participant group consisted of students from the second, third, and fourth grades of primary school. To ensure age-appropriate content and reading complexity, different texts were selected to represent each grade level. All texts were sourced from official textbooks published by the Ministry of National Education of the Republic of Turkey.

To minimize the likelihood that participants had previously encountered the texts, materials were selected from textbooks intended for one grade level above the participant’s current grade. For example, second-grade students were presented with texts extracted from third-grade textbooks. Detailed information about the texts is given in Supplementary Table S1.

Font sizes were chosen to reflect the minimum and maximum values commonly used in Ministry-approved textbooks, ensuring ecological validity. Line spacing was standardized at 2.0 for all texts except for Text 28, which served as a control with different spacing.

During the experiment, each participant was asked to read a total of 28 texts. The texts were displayed sequentially using a PowerPoint presentation. Transitions between texts were manually controlled by a research assistant, who also monitored the participants’ reading progress. Upon completion of each text, the assistant stopped the recording, which was managed by the BIOPAC MP-36 system. The duration of each reading task was automatically recorded by the BIOPAC system, ensuring precise synchronization between the reading activity and physiological data acquisition. Participants were instructed to read aloud at their natural, everyday pace, without the use of any specialized reading techniques. The duration of the experiment varied across participants, depending on individual reading speed.

To mitigate fatigue and maintain attention, a break of 3 to 5 min was provided after every four texts. These sets of four texts shared the same typeface but differed in font size, allowing for controlled comparison within each typeface condition.

### 2.4. Reading Time

**Figure 3:**
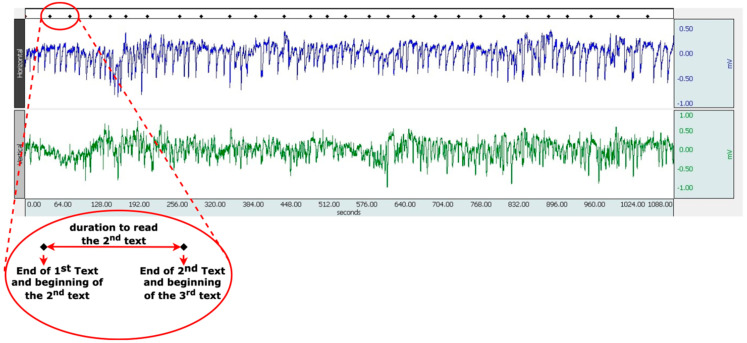
Calculation of reading time using BIOPAC.

Reading times were calculated using the BIOPAC MP36 data recording system. When the reading of each text is finished, the marking is done by the data recording system automatically. Figure 3 shows the determination of text reading times using BIOPAC. The red circled mark in the figure is the mark made after each text reading. Text reading time was determined by calculating the time difference between the two marked points.

### 2.5. Number of Regression

In several studies in the literature, it has been reported that people with dyslexia perform regressions/re-reading during reading more than a normal reader [43,44,45]. Considering this information, determination of the regression movement in EOG signals may be important for the detection of dyslexia. As the eye moves from left to right while reading a text, the amplitude of the EOG signal changes. When we start reading from the beginning of the line (left) until the end of the line (right), eyeballs approach the positive electrode, because of this positive amplitude occurs in the EOG signal. While reading, if the subject returns to the previous or earlier words (to the left), the amplitude of the EOG signal suddenly changes to a negative value. This negative amplitude change creates a regression movement. In our previous study [46], we developed a method that automatically determines the regression movement. We used the same method to determine the number of regression movements in this study.

### 2.6. Number of Blink

**Figure 4:**
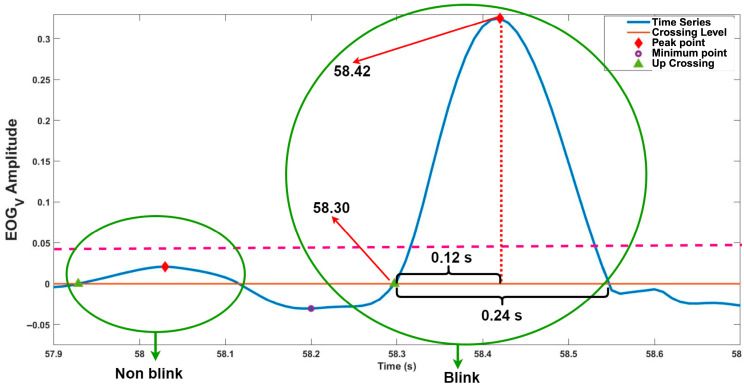
Determination of blink movement.

There are many studies to determine the blink movement by using EOG signals [13,47,48,49,50]. In this study, the number of blinks was determined using vertical EOG signals. First, vertical EOG signals were filtered with a Notch filter to remove 50 Hz noise, and then baseline correction was performed. After pre-processing, the peak, minimum, and up-crossing points of the vertical EOG signal were computed. In the literature, blink duration was determined as 100 ms–800 ms (0.1 s–0.8 s) [50]. In this study, if the time between the up-crossing and the crest point is in the range of 50 ms–400 ms, the peak points of this condition were determined as a blink. In addition, in determining the number of blinks, the threshold amplitude value was determined for each signal, and blink computation was made for the amplitude values above this threshold value (dashed, horizontal, pink line in Figure 4). If the amplitude of the EOG signals is below the threshold, it is not counted as a blink. Figure 4 illustrates the determination of blink movement.

### 2.7. Energy of EOG Signals

EOG signal amplitude shows how far the eyes moved from the reference position and its changes with saccades, blinks, and regression, etc., which are the kinematics of eye movements. Therefore, “energy of EOG signals” relates to the kinetic energy involved in moving the eye from one fixation point to another. The energies of the EOG signals for each text were calculated using Equation (1).

(1) $$\int_{-\infty }^{+\infty }{\left|x\left(t\right)\right|}^{2}dt\;$$

### 2.8. Scalogram Images

In this study, due to the use of 2D CNN models, the one-dimensional EOG signals were transformed into two-dimensional scalogram images to enable effective classification. Continuous wavelet transform (*CWT*) is one of the time–frequency domain conversion methods [51]. It transforms 1-D physiological signals such as EEG [52,53], EMG [54,55], and ECG into 2-D time–frequency spectrums which can be directly used by the convolution layers. Scalogram images were created in MATLAB R2024a using *CWT* at a resolution of 300 DPI. The mathematical formula for calculating *CWT* is expressed as:

(2) $$CWT\left(a,b\right)=\frac{1}{\sqrt{a}}\int_{-\infty }^{\infty }x(t)\psi^{*}(\frac{t-b}{a})dt$$

where *x*(*t*) is the time domain signal, ψ* (*t*) is the complex conjugate wavelet function, *a* is the scale coefficient, and *b* is the time-shift coefficient.

The scalogram images show the amount of energy distribution in the time shift (*b*) and scaling factor (*a*) of EOG signals. It is calculated as the magnitude squared of the continuous wavelet transform, and the formula for computing is given in Equation (3).

(3) $${Energy}_{CWT}\left(a,b\right)={\left|CWT\left(a,b\right)\right|}^{2}$$

**Figure 5:**
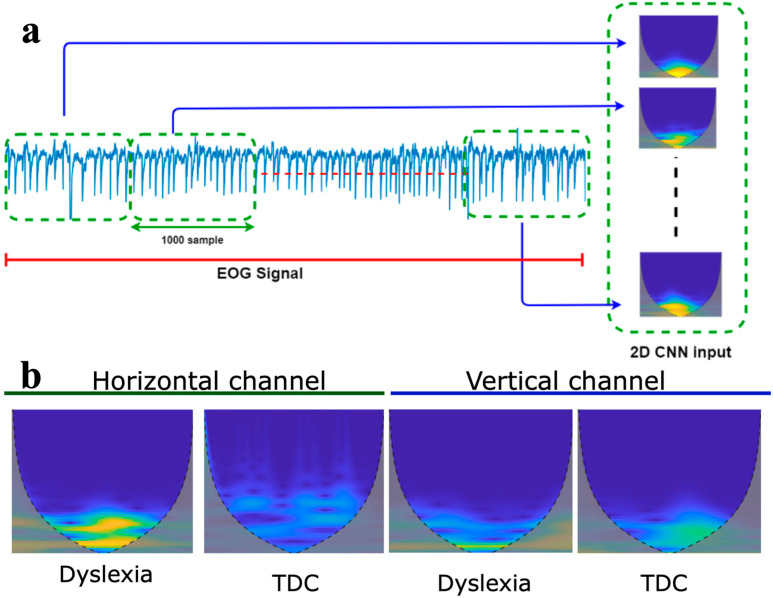
(**a**) The process of converting EOG signals to scalogram signals, (**b**) scalogram of EOG signals: from horizontal channel dyslexia and healthy; from vertical channel dyslexia and typically developing controls.

EOG signals were segmented into signal frames (each frame consists of 1000 EOG samples). Following this, each frame converts into a scalogram image. Figure 5a illustrates the step of converting EOG signals to scalogram signals. 3000 scalogram images were used for both groups in this study. This process is applied to both channel signals. Figure 5b shows the scalogram images of dyslexia and TDC obtained using *CWT* method from the EOG signals recorded from the horizontal and vertical channels.

### 2.9. AlexNet

AlexNet was developed by Alex Krizhevsky, Ilya Sutskever, and Geoffrey Hinton in 2012 [56]. It won the ImageNet Large Scale Visual Recognition Challenge (ILSVRC) in 2012. AlexNet consists of several layers, including 5 convolutional layers, 3 fully connected layers, 5 max pooling layers, and one SoftMax layer. Apart from these layers, it has RELU and dropout layers. The input data size should be 227 × 227 × 3 for AlexNet.

### 2.10. ResNet50

ResNet (Residual Neural Network) [57] is specifically designed to address issues related to deepening traditional deep networks. To overcome these problems, it utilizes “skip connections”; that is, it directly adds the outputs of previous layers to the outputs of later layers. These connections facilitate the learning process of the network and stabilize the training of deeper networks.

### 2.11. MobileNet

MobileNet is an artificial neural network architecture developed by Google for computer vision tasks based on deep learning [58]. It is designed to provide efficient and lightweight modeling in resource-constrained environments such as mobile devices. MobileNet employs various techniques to optimize deep neural network architectures, particularly utilizing a structure called “depth-wise separable convolution” to significantly reduce computational costs while decreasing the model’s depth. This enables the creation of faster and lighter models while maintaining high levels of accuracy.

### 2.12. DyslexiaNet

**Figure 6:**
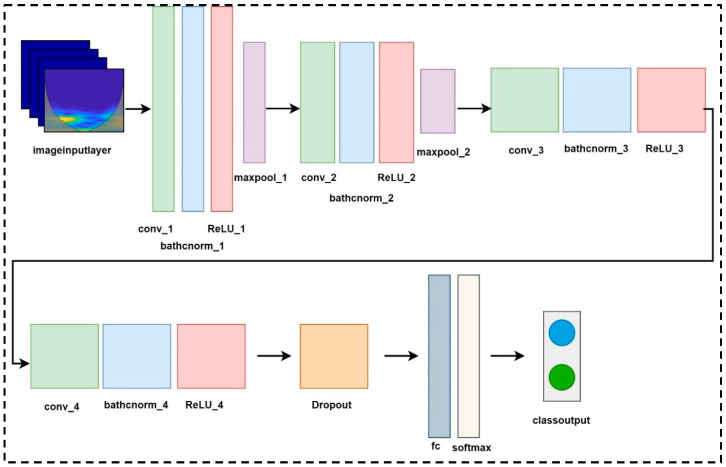
Architecture of DyslexiaNet.

A summary of the DyslexiaNet model framework is presented in Table 1. The presented model employs four convolution layers with other layers. The first CNN layer processes the input scalogram images with a size of 28 × 28 × 3. The first convolutional layer (Conv) has 16 filters of size with a stride of 1 and the same padding. Following the first Conv1, the batch normalization and ReLU layers are applied. Next, a 2 × 2-sized 2D max pooling layer with a stride of 2 is applied. This structure repeated 4 times with different sizes of filters and strides. The second, third, and fourth Conv layers have 32, 64, and 64 filters of size, and each layer maintains the same padding with a stride value of 1. The dropout layer used a value of 0.5 to prevent overfitting. The SoftMax layer is the last layer of the proposed network to predict the class of an input image. The proposed DyslexiaNet model is illustrated in Figure 6. Hyperparameters of the DyslexiaNet model are given in Supplementary Table S3.

## 3. Results

### 3.1. Children with Dyslexia Have a Higher Reading Time

**Figure 7:**
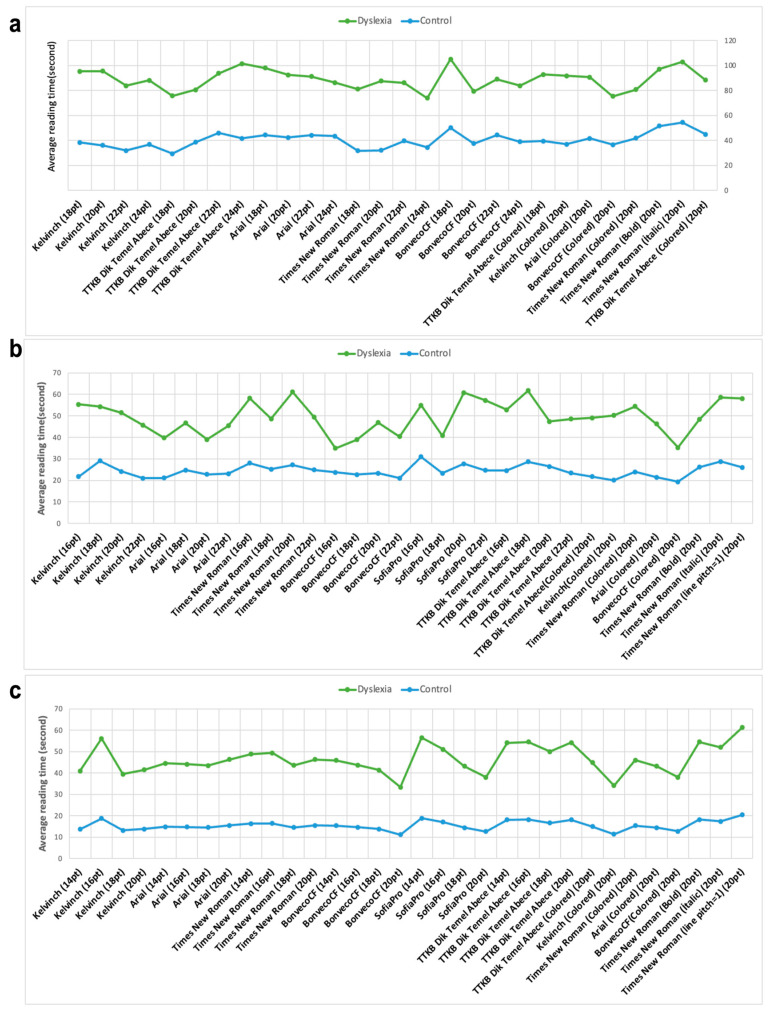
The average reading time results for second (**a**), third (**b**) and fourth (**c**) grades subjects. The *x*-axis represents text information, and the *y*-axis represents average reading time in second.

**Figure 8:**
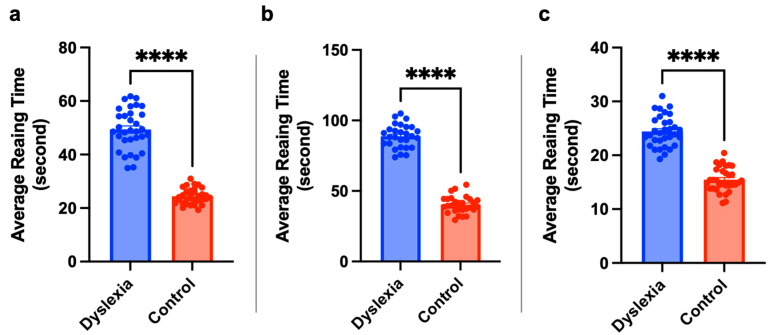
Children with dyslexia have significantly higher reading times. The statistical results for second (**a**), third (**b**) and fourth (**c**) grades subjects with independent sample *t*-test, ****: *p* < 0.0001. Statistical analysis was performed using GraphPad Prism 10.0^®^ software.

The average reading time of each text was calculated for each group separately. Then, we analyzed whether there was a significant difference between individuals with dyslexia and TDC individuals in terms of average reading time. Figure 7 shows the average reading time of the texts of individuals with dyslexia and TDC individuals. Analysis of reading times among dyslexic students across grade levels revealed distinct preferences for specific typeface and font size combinations. For second-grade students with dyslexia, the shortest average reading times were observed with texts formatted in TTKB Dik Temel ABC at 18 points (pt), Times New Roman at 16 pt, and BonvenoCF-Colored at 20 pt. In the third grade, BonvenoCF at 16 pt yielded the fastest reading time (34.956 s), followed closely by BonvenoCF-Colored at 20 pt (35.252 s). Across both second and third grades, texts presented in italicized fonts consistently resulted in longer reading times, suggesting that italic styling may hinder reading fluency in dyslexic children. Among fourth-grade students, a gradual decrease in average reading time was noted across texts 13 to 16, all of which were prepared using the BonvenoCF typeface. Notably, the 16th text, formatted in BonvenoCF at 20 pt, produced the lowest average reading time (33.324 s), indicating that this combination may be particularly effective for older dyslexic readers. Statistical analysis results are shown in Figure 8. The reading time of individuals with dyslexia is significantly higher than the reading time of TDC.

### 3.2. Children with Dyslexia Tend to Blink More

**Figure 9:**
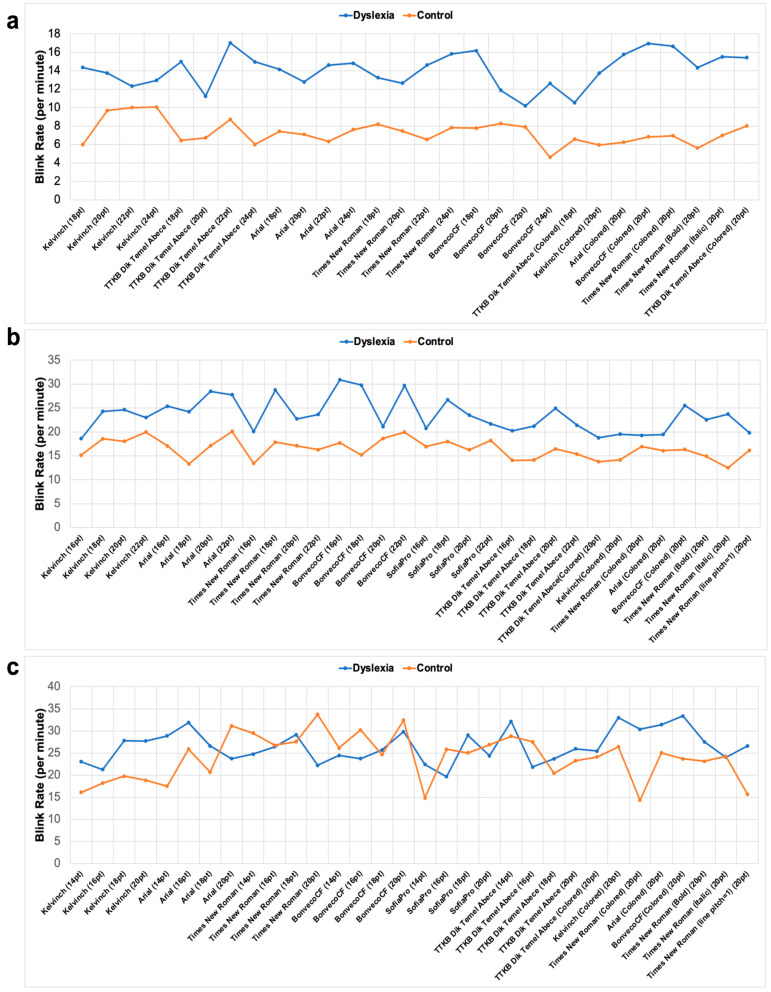
The blink rate results for second (**a**), third (**b**), and fourth (**c**) grade subjects. The *x*-axis represents text information, and the *y*-axis represents blink rate. The blink rate was calculated by dividing the average number of blinks in each text by the reading time in minutes of that text.

**Figure 10:**
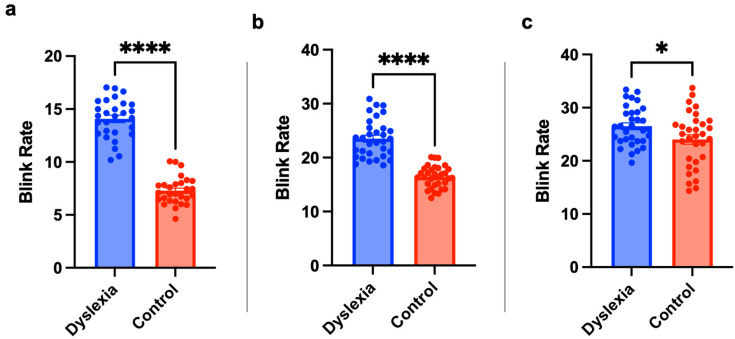
Children with dyslexia have significantly higher blink rate. The statistical results for second (**a**), third (**b**), and fourth (**c**) grades subjects with independent sample *t*-test, ****: *p* < 0.0001, *: *p* < 0.05. Statistical analysis was performed using GraphPad Prism 10.0^®^ software.

Another feature obtained from EOG signals was the blink rate. The blink rate of each text was calculated for each group separately. Figure 9 shows the blink rate of the texts of students with dyslexia and the TDC group. As can be seen from the figure, the blink rate of individuals with dyslexia is higher than TDC group. Blink rate during reading was analyzed as an indicator of cognitive load and visual strain across dyslexic students in the second, third, and fourth grades. Among second-grade participants, the lowest blink rate was recorded during the reading of the text formatted in BonvenoCF at 22 pt. For third-grade students, the Times New Roman at 16 pt BonvenoCF at 20 pt, SofiaPro 16 pt and TTKB Dik Temel ABC elicited the fewest blinks, indicating a similar trend in reduced cognitive demand. In the fourth-grade cohort, like third grade students Times New Roman at 20 pt, SofiaPro at 16 pt and TTKB Dik Temel ABC showed lower blink rate in dyslexia group. However, surprisingly, TDC groups showed a higher blink rate in some typefaces such as BonvenoCF compared to the dyslexia group. Statistical analysis results are shown in Figure 10. Although the results showed that the dyslexia group had a significantly higher blink rate than the TDC group, the blink rate in fourth grades was not as different as it was in second and third grades, where a big difference was seen between the dyslexia and TDC groups.

### 3.3. The Regression Rate Is Significantly Higher in the Dyslexia Group

**Figure 11:**
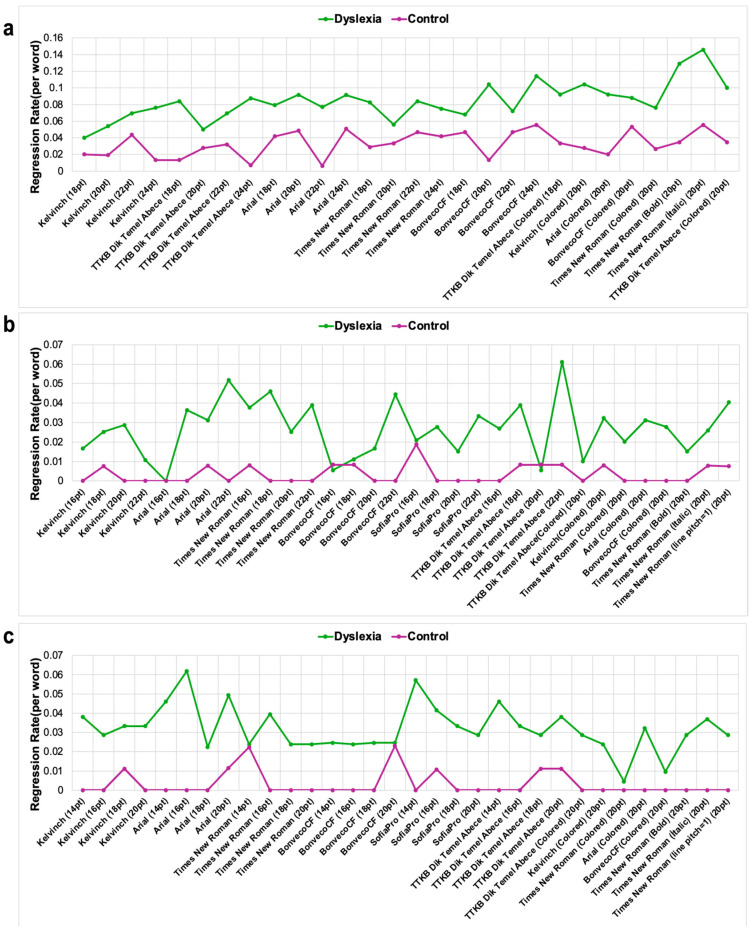
The regression rate results for second (**a**), third (**b**), and fourth (**c**) grade subjects. The *x*-axis represents text information, and the *y*-axis represents regression rate. The regression rate was calculated by dividing the average number of regressions by the number of words in the text.

**Figure 12:**
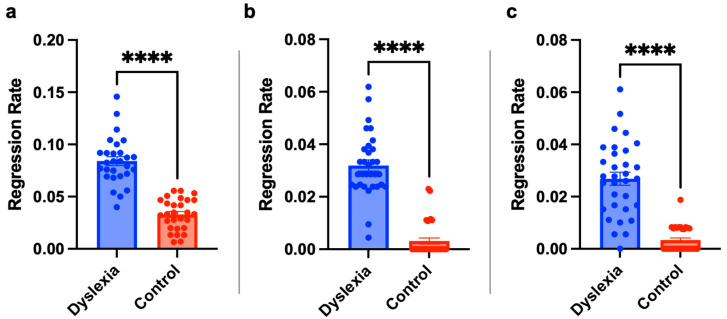
Children with dyslexia have a significantly higher regression rate. The statistical results for second (**a**), third (**b**), and fourth (**c**) grades subjects with independent sample *t*-test ****: *p* < 0.0001. Statistical analysis was performed using GraphPad Prism 10.0^®^ software.

The number of regressions from EOG signals was determined using the method specified in Section 2.4. Like other features, the regression rate of each text was calculated for each group separately, and statistical evaluations were made. Figure 11 depicts the regression rates of the texts of individuals with dyslexia and the TDC group. Second-grade students exhibited reduced regression when reading tasks presented in TTKB Dik Temel Abece at 20 pt and Times New Roman at 20 pt, suggesting that larger font sizes and specific typefaces may facilitate smoother reading for younger dyslexic readers in terms of regression. Among third-grade students, lower regression rates were observed with BonvenoCF at 16 pt, TTKB Dik Temel Abece at 20 pt, and Arial at 16 pt. For fourth-grade students, Times New Roman at 14 pt and a colored variant of Times New Roman at 20 pt were associated with reduced regression. This suggests that both font size and visual enhancements (e.g., color differentiation) may play a role in mitigating reading difficulties. Notably, BonvenoCF demonstrated consistently low regression across font sizes ranging from 14 pt to 20 pt, outperforming other typefaces in terms of stability and effectiveness. This consistency highlights BonvenoCF as a potentially optimal typeface for dyslexic readers across multiple grade levels. Statistical test results were given in Figure 12. Test results revealed that children with dyslexia have a significantly higher regression rate compared to healthy subjects.

### 3.4. The Energy of EOG Signals Shows an Increase in the Dyslexia Group

**Figure 13:**
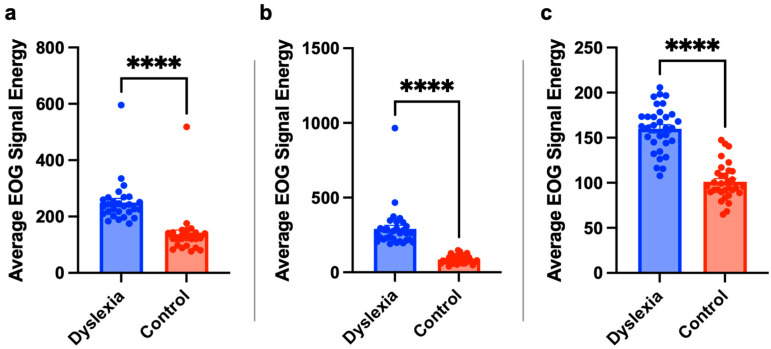
Children with dyslexia have significantly higher EOG signal energy. The statistical results for second (**a**), third (**b**) and fourth (**c**) grades subjects with independent sample *t*-test ****: *p* < 0.0001. Statistical analysis was performed using GraphPad Prism 10.0^®^ software.

The energy of horizontal EOG signals was computed for all texts. In terms of average EOG signal energies for each text, whether there is a significant difference between individuals with dyslexia and TDC was analyzed. The statistical test results are given in Figure 13, where children with dyslexia have significantly higher EOG signal energy than TDC.

### 3.5. Classification Results

As mentioned in Section 2, we proposed a new simple deep learning architecture to classify our scalogram images converted from the EOG signal. AlexNet, ResNet50, MobileNetV2, and DyslexiaNet models were used for the classification of healthy and dyslexic scalogram images obtained from EOG signals. The CNN models were used to classify both horizontal and vertical channel EOG scalograms separately. Two different channels are described in Supplementary Table S4. Three thousand scalograms were used in both groups with k-fold cross-validation. The performance of each fold was computed separately, and the final performance was determined by averaging over all k-fold results. In this study, K was set at 5. The performance of networks was evaluated using accuracy, sensitivity, specificity, and F1-score performance metrics. The confusion matrix (CM) was used to calculate these metrics.

(4) $$Accuracy=\frac{\left(tp+tn\right)}{\left(tp+tn+fp+fn\right)}$$

(5) $$Sensitivity=\frac{tp}{tp+fn}$$

(6) $$Specificity=\frac{tn}{\left(tn+fp\right)}$$

(7) $$\mathrm{F1-Score}=\frac{2tp}{2tp+fp+fn}$$

**Figure 14:**
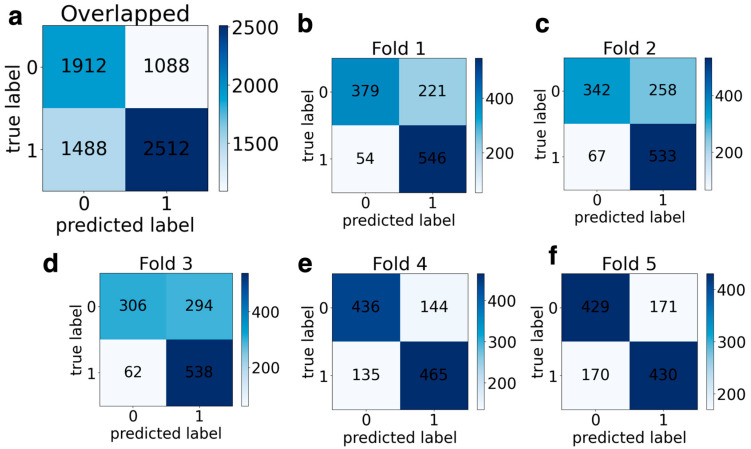
Channel 1 overlapping and 5-fold confusion matrices for DyslexiaNet: (**a**) overlapped CM, (**b**) Fold 1 CM, (**c**) Fold 2 CM, (**d**) Fold 3 CM, (**e**) Fold 4 CM, (**f**) Fold 5 CM.

Table 2 shows the classification performance results for channel 1. The accuracy, sensitivity, specificity, and F1-score values, as well as mean (±standard deviation) values, are given for 5-fold cross-validation. The average accuracy of networks was 65.61%, while the average values of sensitivity, specificity, and F1-score results for AlexNet in the vertical channel were obtained at 54.43%, 76.8%, and 60.97%, respectively. The overlapped and 5-fold confusion matrix for the vertical channel is given in Supplementary Figure S2. AlexNet correctly classified 3937 out of 6000 scalogram images and achieved an overall accuracy of 65.61% for the vertical channel. When it comes to ResNet50, average values of sensitivity, specificity, and F1-score were 55.56%, 51.13%, and 4.95%, respectively, and accuracy was 53.35%. The overlapped and 5-fold confusion matrix for the vertical channel is given in Supplementary Figure S3. ResNet50 correctly classified 3201 out of 6000 scalogram images. For MobileNetV2, average results were 57.01%, 42.61%, 71.44%, and 49.70%, respectively, for accuracy, sensitivity, specificity, and F1-score. The overlapped and 5-fold confusion matrix for the vertical channel is given in Supplementary Figure S4. MobileNetV2 correctly classified 3421 out of 6000 scalogram images. For DyslexiaNet, average values of accuracy, sensitivity, specificity, and F1-score were 73.73%, 63.73%, 83.72%, and 70.50%, respectively. The confusion matrix is presented in Figure 14.

**Figure 15:**
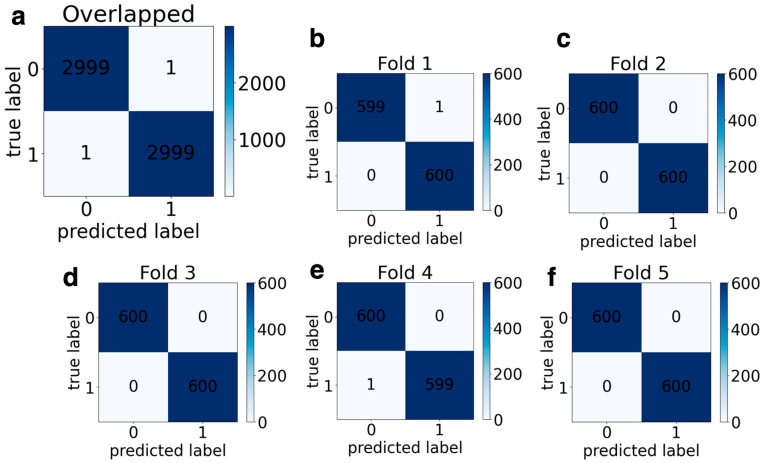
Channel 2 overlapping and 5-fold confusion matrices for DyslexiaNet: (**a**) overlapped CM, (**b**) Fold 1 CM, (**c**) Fold 2 CM, (**d**) Fold 3 CM, (**e**) Fold 4 CM, (**f**) Fold 5 CM.

**Figure 16:**
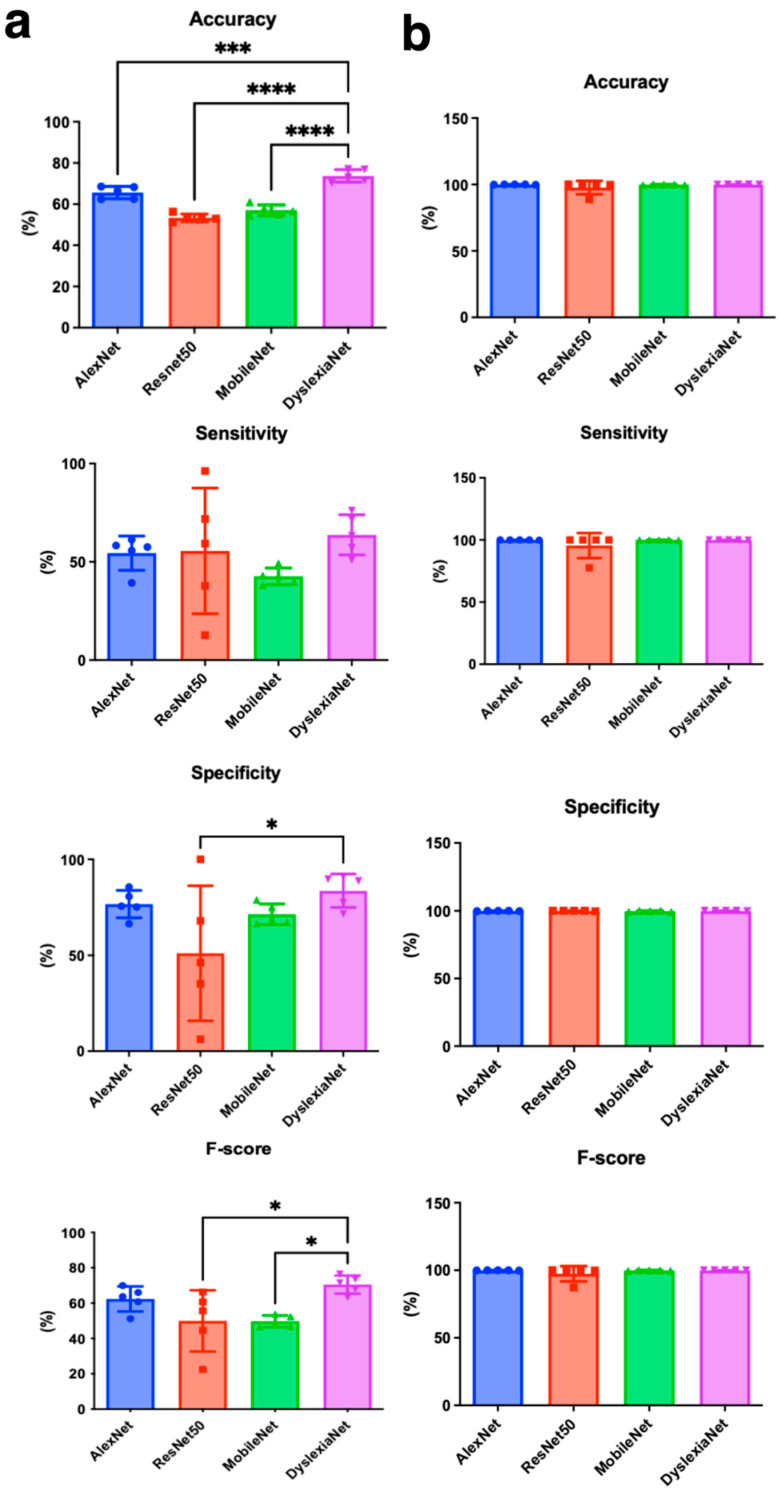
The comparison of classification metrics for (**a**) vertical and (**b**) horizontal EOG with One Way ANOVA with Dunnett correction: accuracy, sensitivity, specificity, F-score, * *p* < 0.05, *** *p* < 0.0001, **** *p* < 0.00001. Statistical analysis was performed using GraphPad Prism 10.0^®^ software.

Table 3 presents the performance of channel 2 classification results. The AlexNet model achieved an average accuracy of 99.94%, while the average sensitivity, specificity, and F1-score for the horizontal channel were 99.96%, 99.92%, and 99.94%, respectively. In terms of ResNet50 results, the average scores of all folds were 97.71%, 95.00%, 99.93%, and 97.43%, respectively, for accuracy, sensitivity, specificity, and F1-score. For MobileNetV2, average values of accuracy, sensitivity, specificity, and F1-score were obtained at 99.80%, 99.63%, 99.63%, and 99.78%, respectively. The overlapped and 5-fold confusion matrix for horizontal channels is given in Supplementary Figures S5–S7 for AlexNet, ResNet50, and MobileNetV2, respectively. Figure 15 shows a confusion matrix for overlapping and a 5-fold for DyslexiaNet. The AlexNet, ResNet50, and MobileNetV2 were correctly classified as 5997, 5863, and 5988 out of 6000 scalogram images, respectively. When it comes to DyslexiaNet, it reached 99.96% classification accuracy. The other classification metric of DyslexiaNet was 99.96% for sensitivity, specificity, and F1-score. The comparison of all performance metrics for all networks is given in Figure 16a,b for vertical and horizontal channels, respectively.

**Figure 17:**
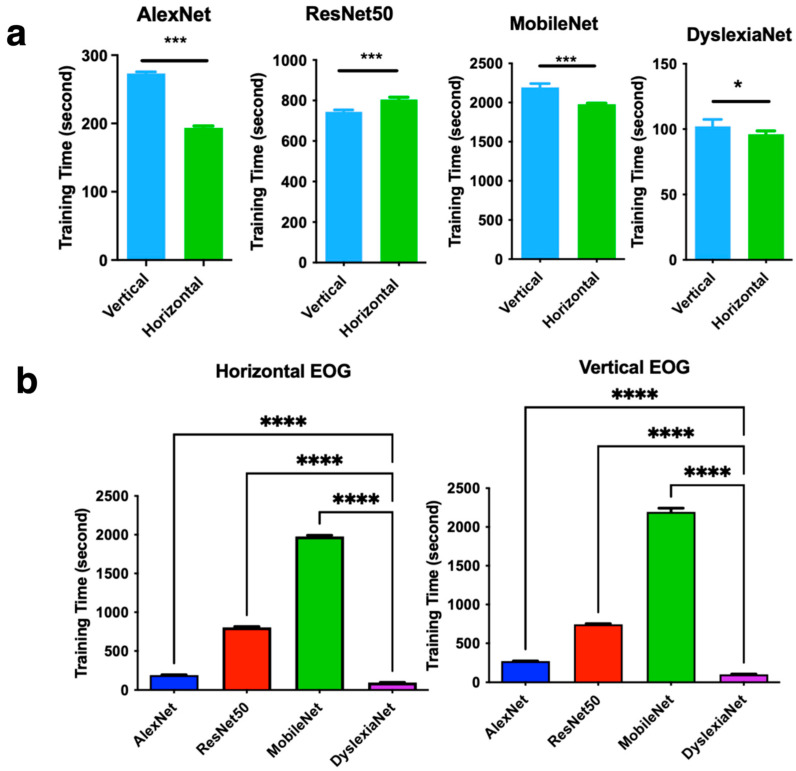
Average training time comparison for CNN methods with unpaired *t*-test with Welch’s correction for (**a**) AlexNet, ResNet50, MobileNet, DyslexiaNet, (**b**) average training time for CNN methods with One Way ANOVA with Dunnett correction for AlexNet, ResNet50, MobileNet, DyslexiaNet, * *p* < 0.05, *** *p* < 0.0001, **** *p* < 0.00001. Statistical analysis was performed using GraphPad Prism 10.0^®^ software.

To test the computational workload, we also calculated the training time of the CNN models. The longer training time can increase the computational load for the machine and can affect the results. We looked at the training time for each network separately for both channels in Figure 17a. The vertical channel training time was significantly higher than the horizontal channel for AlexNet, MobileNet, and DyslexiaNet, whereas for ResNet50, it was significantly lower. We also compared the training times of networks for both vertical and horizontal EOG signals. The results are presented in Figure 17b. The mean training time of the horizontal channel was recorded as 193, 804, 1978, and 96 s for AlexNet, ResNet50, MobileNet, and DyslexiaNet, respectively. AlexNet, ResNet50, MobileNet, and DyslexiaNet have slightly longer training times for the vertical channel, with 273, 744, 2195, and 102 s of training time, respectively. The results provide further insights into the performance differences among the tested deep learning models. The results reveal significant differences in computational efficiency across models for both horizontal and vertical electrooculography channels.

## 4. Discussion

### 4.1. Typeface and Reading Performance in Dyslexia

Considering there is no study to determine the most suitable typeface by using EOG signals, this study aims to determine the most appropriate writing characteristics for use in education to prevent negativity arising from the lack of individualized learning material in the education of children with dyslexia. One of the most widely accepted characteristics of dyslexia is a slower reading speed compared to that of typically developing readers. Accordingly, the first parameter examined in this study was reading time. The results confirmed that children with dyslexia consistently required more time to read texts than their non-dyslexic peers, aligning with previous findings associating dyslexia with slower and more effortful reading. Notably, among the various typefaces tested, BonvenoCF was associated with the shortest average reading time for dyslexic readers, suggesting that font design can significantly influence reading efficiency. In contrast, the TDC group exhibited minimal variation in reading time across different texts, indicating that font style had less impact on fluent readers.

Among the typefaces examined, Times New Roman, BonvenoCF, and TTKB Dik Temel Abece—the official font used in Ministry of National Education textbooks—emerged as particularly influential in shaping reading performance among dyslexic students. Second-grade students demonstrated improved reading efficiency with TTKB Dik Temel Abece and BonvenoCF, likely due to their familiarity with TTKB Dik Temel Abece in school materials. Across other grade levels, BonvenoCF consistently yielded better reading performance, particularly in third-grade students. Additionally, using colored syllables with BonvenoCF supported syllable-level decoding, reducing cognitive load and improving visual parsing.

### 4.2. Blink Behavior and Regression

In addition to reading time, blink behavior was analyzed as a potential physiological marker of reading difficulty. Statistical analysis revealed a significant difference in the average number of blinks between dyslexic and non-dyslexic children, with dyslexic readers exhibiting a higher blink rate during reading tasks. This supports the hypothesis that blink frequency may reflect increased cognitive load or visual stress in dyslexic individuals and could serve as a supplementary diagnostic indicator.

Another commonly held belief about dyslexia is that affected individuals tend to read words backward or revisit previously read text. This behavior, known as regression, was quantitatively assessed using eye-tracking data. The results demonstrated that children with dyslexia performed significantly more regression movements than their non-dyslexic counterparts across all font sizes and grade levels. These findings are consistent with prior research indicating that dyslexic readers exhibit more frequent regressive saccades and longer fixation durations, reflecting difficulties in word decoding and comprehension.

### 4.3. EOG Signal Energy and Physiological Markers

To further investigate the physiological correlates of reading difficulty, EOG signal energy was analyzed. EOG signals increase in amplitude during eye movements such as blinking and regression. Therefore, lower EOG signal energy during reading is interpreted as an indicator of smoother and less effortful reading. The study found statistically significant differences in EOG signal energy between dyslexic and non-dyslexic children across all groups, with dyslexic readers showing higher energy levels. These results suggest that EOG signal energy may be a valuable biomarker for assessing reading difficulty and could enhance the accuracy of dyslexia diagnosis when combined with behavioral metrics.

### 4.4. Methodological Considerations and Previous Work

Traditional research has yet to effectively determine the role of cognitive skills in reading problems, probably because reading involves multiple interacting components that conventional statistical approaches cannot fully capture. As a result, the number of research projects utilizing artificial intelligence (AI) and CNN methodologies has increased. Previous research has used a variety of data sources using machine learning algorithms to diagnose dyslexia, including MRI images [59], fMRI images [37], EEG signals [60], games [61,62], reading errors [63,64], facial images [65], eye movements [41,62,66], and handwriting [67,68]. Taş et al. [50] developed a machine learning model to predict dyslexia in Turkish-speaking children using audio recordings, achieving a high accuracy of 95.63% [69]. While this study is one of the rare studies in Turkish children, it differs from the present study in that voice signals were used instead of EOG signals, and the methodology was completely different.

We conducted pilot studies in Turkish to investigate the relationship between EOG signals and reading tasks. In our previous work [70], ten TDC subjects read a Turkish text in Times New Roman at 12 pt. EOG signals were recorded to identify retrieving words/re-reading and skipping line movements, which were then used as features for classifiers, achieving 98% classification accuracy with Random Forest and k-NN. This was later expanded to include children with dyslexia [46], with retrieving words/re-reading movements detected at 97.11% and skipping line movements at 93.96% success.

A preliminary study [71] tested whether reading performance changes when typefaces are modified. Using Times New Roman in four font sizes (16, 18, 20, and 22 pt) and horizontal EOG signals from 20 subjects, a 1D CNN classifier achieved 73.61% classification accuracy between TDC and dyslexic children. In addition, in our recent study [38], EOG signals from horizontal and vertical channels were evaluated separately, achieving 98.70% and 80.94% accuracy, respectively. The current study extends this work by using extracted features from EOG signals for typeface selection, rather than solely dyslexia classification.

### 4.5. Deep Learning Approach and Network Performance

We proposed a novel approach using scalogram images converted from EOG signals as input for three well-known CNN methods—AlexNet, MobileNet, ResNet—and our proposed network, DyslexiaNet. Both horizontal and vertical channels were analyzed separately. Horizontal movements capture reading-related saccades, fixations, and regressions, while vertical movements are relevant for line transitions. DyslexiaNet demonstrated slightly better performance for the horizontal channel and significantly higher accuracy for the vertical channel than other networks. The horizontal channel consistently outperformed the vertical channel due to its greater sensitivity to reading-related eye movements.

Supplementary Table S5 summarizes the fundamental parameters of all the networks used in this study. Training time analysis showed that DyslexiaNet required the shortest time for both channels, followed by AlexNet. ResNet50 and MobileNetV2 had longer training times. DyslexiaNet’s simpler architecture with only 4 convolutional layers achieved high accuracy while minimizing computational load, avoiding potential overfitting seen in deeper networks, especially for vertical channel data. Also, when we compared DyslexiaNet performance versus our previous study [38], where we used 1D-CNN, we increased the classification accuracy using DyslexiaNet to 99.96% for the horizontal channel and 96.70% for the 1D-CNN.

### 4.6. Advantages and Implications

The main advantages of this proposed method are as follows:

- The proposed method is a non-invasive and objective method using the EOG signals in children with dyslexia to determine the best typeface for them.
- Since more than one typeface and font (28 texts in seven different typefaces and four different font sizes) are used, it provides a more general evaluation. A single typeface will not be sufficient to reach a general conclusion.
- A new deep neural network model was proposed to detect dyslexia using scalogram images of EOG signals recorded while reading tasks in different typefaces and fonts in Turkish-speaking children.
- The proposed method is easy to use and can be applied in real time.

These findings highlight the potential for integrating physiological and behavioral indicators to guide individualized educational strategies and support clinicians in the early detection of dyslexia.

## 5. Conclusions

In conclusion, the most suitable typefaces for children with dyslexia were determined by using EOG signals in this study. In contrast to common typefaces such as Arial, our results showed that an increase in reading speed and fewer reading mistakes were seen in BonvenoCF; however, each child could differ individually, and features such as blinking and re-reading were distinctive between children with dyslexia and the TDC group. Thus, by determining which typeface is proper for children diagnosed with dyslexia to read more easily, faster, and better, the educational life of the child could be improved by preparing individual materials.

Also, features can be used to support the diagnosis of dyslexia apart from the specific learning disability battery. A data-based method has been developed by analyzing EOG signals that evaluate dyslexia and eliminate subjectivity compared to the specific learning disability battery used in the clinic. The proposed method is a non-invasive and objective method using EOG signals in individuals with dyslexia. The classification results obtained from EOG signals showed higher classifier accuracy in the prediction of dyslexia. This proposed method will be implemented as a decision support system that helps physicians.

## 6. Limitations and Future Work

This study has some limitations. First, the relatively limited sample size and the restriction of participants to a specific age and language group limit the generalizability of the findings. In addition, the data set and modeling methods used in the study may not fully reflect the multidimensional and complex nature of dyslexia. Future studies plan to incorporate broader and more heterogeneous sample groups, enable multimodal data integration, and conduct long-term follow-up studies. Also, this study primarily aimed to evaluate deep learning–based dyslexia detection and simply provide some main features, i.e., reading time, regression rate, etc., to evaluate typefaces and font effects. Typeface findings were exploratory, as pairwise statistical comparisons between fonts were not performed, and text content was not strictly controlled. Future research should employ standardized texts and controlled font conditions to draw firmer conclusions.

## Figures and Tables

**Table 1:** Summary of the DyslexiaNet model.

#	Name	Type	Activations	Learnables		Total Learnables
1	imageinput 28 × 28 × 3 images with ‘zerocenter’ normalzation	Image Input	28 × 28 × 3	-		0
2	Conv_1 16- 4 × 4 × 3 convolutions with stride [1 1] and padding ‘same’	Convolution	28 × 28 × 16	Weights Bias	4 × 4 × 3 × 16 1 × 1 × 16	784
3	Batchnorm_1 Batch normalization with 8 channels	Batch Normalization	28 × 28 × 8	Offset Scale	1 × 1 × 16 1 × 1 × 16	32
4	Relu_1 ReLU	ReLU	28 × 28 × 16	-		0
5	Maxpool_1 2 × 2 max pooling with stride [2 2] and padding [0 0 0 0]	Max Pooling	7 × 7 × 16	-		0
6	Conv_2 32 3 × 3 × 8 convolutions with stride [1 1] and padding ‘same’	Convolution	7 × 7 × 32	Weights Bias	4 × 4 × 16 × 32 1 × 1 × 32	8224
7	Batchnorm_2 Batch normalization with 16 channels	Batch Normalization	7 × 7 × 32	Offset Scale	1 × 1 × 32 1 × 1 × 32	64
8	Relu_2 ReLU	ReLU	7 × 7 × 32	-		0
9	maxpool_2 2 × 2 max pooling with stride [2 2] and padding [0 0 0 0]	Max Pooling	3 × 3 × 32	-		0
10	conv 3 64 3 × 3 × 16 convolutions with stride [1 1] and padding ‘same’	Convolution	3 × 3 × 64	Weights Bias	4 × 4 × 32 × 64 1 × 1 × 64	32,832
11	batchnorm 3 Batch normalization with 32 channels	Batch Normalization	3 × 3 × 64	Offset Scale	1 × 1 × 64 1 × 1 × 64	128
12	relu 3 ReLU	ReLU	3 × 3 × 64	-		0
13	Conv_4 64 3 × 3 × 32 convolutions with stride [1 1] and padding ‘same’	Convolution	3 × 3 × 64	Weights Bias	4 × 4 × 64 × 64 1 × 1 × 64	65,600
14	batchnorm 4 Batch normalization with 32 channels	Batch Normalization	3 × 3 × 64	Offset Scale	1 × 1 × 64 1 × 1 × 64	128
15	relu 4 ReLU	ReLU	3 × 3 × 64	-		0
16	Dropout 50% dropout	Dropout	3 × 3 × 64	-		0
17	fc 2 fully connected layers	Fully Connected	1 × 1 × 2	Weights Bias	2 × 576 2 × 1	1154
18	SoftMax SoftMax	Softmax	1 × 1 × 2	-		8
19	Classoutput crossentropyex	Classification Output		-		0

**Table 2:** Performance results for the five folds for the vertical channel.

CNN Model	Fold Number	Accuracy (%)	Sensitivity (%)	Specificity (%)	F1-Score (%)
**AlexNet**	Fold-1	62.50	39.33	85.67	51.19
	Fold-2	68.25	55.67	80.83	63.68
	Fold-3	68.50	61.33	75.67	66.07
	Fold-4	66.42	57.50	75.33	69.98
	Fold-5	62.42	58.33	66.50	60.82
	**Mean ± Std.**	**65.61 ± 2.67**	**54.43 ± 7.76**	**76.80 ± 6.39**	**60.97 ± 5.16**
**ResNet50**	Fold-1	56.33	12.67	100	22.49
	Fold-2	52.92	37.83	68.00	44.55
	Fold-3	52.83	59.33	46.33	55.71
	Fold-4	53.50	71.83	35.17	60.70
	Fold-5	51.17	96.17	6.17	66.32
	**Mean ± Std.**	**53.35 ± 1.87**	**55.56 ± 31.94**	**51.13 ± 35.25**	**49.95 ± 17.32**
**MobileNetV2**	Fold-1	54.42	39.83	69.00	46.63
	Fold-2	55.08	42.80	67.30	48.80
	Fold-3	56.66	38.20	75.20	46.80
	Fold-4	57.91	49.24	66.70	53.90
	Fold-5	61.00	43.00	79.00	52.40
	**Mean ± Std.**	**57.01 ±2.61**	**42.61 ± 4.22**	**71.44 ± 5.40**	**49.70 ± 3.30**
**DyslexiaNet**	Fold-1	77.08	63.17	91	73.38
	Fold-2	72.92	57	88.83	67.79
	Fold-3	70.33	51	89.67	63.22
	Fold-4	76.75	76	77.5	76.57
	Fold-5	71.58	71.5	71.62	71.56
	**Mean ± Std.**	**73.732 ± 3.04**	**63.734 ± 10.22**	**83.724 ± 8.65**	**70.504 ± 5.16**

**Table 3:** Performance results for the five folds for the horizontal channel.

CNN Model	Fold Number	Accuracy (%)	Sensitivity (%)	Specificity (%)	F1-Score (%)
**AlexNet**	Fold-1	99.91	100	99.80	99.90
	Fold-2	100	100	100	100
	Fold-3	99.83	99.80	99.80	99.80
	Fold-4	100	100	100	100
	Fold-5	100	100	100	100
	**Mean ± Std.**	**99.94 ± 0.068**	**99.96 ± 0.008**	**99.92 ± 0.097**	**99.94 ± 0.008**
**ResNet50**	Fold-1	100	100	100	100
	Fold-2	99.83	100	99.67	99.83
	Fold-3	100	100	100	100
	Fold-4	88.75	77.50	100	87.32
	Fold-5	100	100	100	100
	**Mean ± Std.**	**97.71 ± 5.0127**	**95.00 ± 10.0623**	**99.93 ± 0.1476**	**97.43 ± 5.6521**
**MobileNetV2**	Fold-1	99.92	100	99.83	99.92
	Fold-2	99.58	100	99.17	99.59
	Fold-3	99.58	100	99.17	99.59
	Fold-4	100	100	100	100
	Fold-5	99.92	99.83	100	99.92
	**Mean ± Std.**	**99.80 ± 0.2035**	**99.96 ± 0.0760**	**99.63 ± 0.4292**	**99.80 ± 0.1981**
**DyslexiaNet**	Fold-1	99.92	99.83	100	**99.92**
	Fold-2	100	100	100	**100**
	Fold-3	100	100	100	100
	Fold-4	99.92	100	99.83	99.92
	Fold-5	100	100	100	100
	**Mean ± Std.**	**99.968 ± 0.04**	**99.966 ± 0.07**	**99.966 ± 0.07**	**99.968 ± 0.04**

## Data Availability

Data supporting the study’s findings are accessible from the corresponding author upon reasonable request.

## Supplementary Materials

The following supporting information can be downloaded at: https://www.mdpi.com/article/10.3390/jemr18050056/s1, Table S1: Detailed information about the texts; Table S2: Subject’s characteristics; Figure S1: A summary of the subjects according to their health status; Table S3: Hyperparameter of the DyslexiaNet; Table S4: Definition of two different channels; Figure S2: Channel 1 overlapping and 5-fold confusion matrices for AlexNet: (a) overlapped CM, (b) Fold 1 CM, (c) Fold 2 CM, (d) Fold 3 CM, (d) Fold 4 CM, (f) Fold 5 CM; Figure S3: Channel 1 overlapping and 5-fold confusion matrices for ResNet50: (a) overlapped CM, (b) Fold 1 CM, (c) Fold 2 CM, (d) Fold 3 CM, (d) Fold 4 CM, (f) Fold 5 CM; Figure S4: Channel 1 overlapping and 5-fold confusion matrices for MobileNet: (a) overlapped CM, (b) Fold 1 CM, (c) Fold 2 CM, (d) Fold 3 CM, (d) Fold 4 CM, (f) Fold 5 CM; Figure S5: Channel 2 overlapping and 5-fold confusion matrices for AlexNet: (a) overlapped CM, (b) Fold 1 CM, (c) Fold 2 CM, (d) Fold 3 CM, (d) Fold 4 CM, (f) Fold 5 CM; Figure S6: Channel 2 overlapping and 5-fold confusion matrices for ResNet50: (a) overlapped CM, (b) Fold 1 CM, (c) Fold 2 CM, (d) Fold 3 CM, (d) Fold 4 CM, (f) Fold 5 CM; Figure S7: Channel 2 overlapping and 5-fold confusion matrices for MobileNet: (a) overlapped CM, (b) Fold 1 CM, (c) Fold 2 CM, (d) Fold 3 CM, (d) Fold 4 CM, (f) Fold 5 CM; Table S5: The fundamental parameters of networks.

## References

[1] Catts H.W., Terry N.P., Lonigan C.J., Compton D.L., Wagner R.K., Steacy L.M., Farquharson K., Petscher Y. (2024). Revisiting the definition of dyslexia. Ann. Dyslexia.

[2] McDowell M. (2018). Specific learning disability. J. Paediatr. Child Health.

[3] Wajuihian S.O., Naidoo K.S. (2011). Dyslexia: An overview. Afr. Vis. Eye Health.

[4] Lim W.W., Yeo K.J., Handayani L. (2023). A systematic review on interventions for children with dyslexia. Int. J. Eval. Res. Educ..

[5] Miciak J., Fletcher J.M. (2020). The Critical Role of Instructional Response for Identifying Dyslexia and Other Learning Disabilities. J. Learn. Disabil..

[6] Rice M., Gilson C.B. (2023). Dyslexia Identification: Tackling Current Issues in Schools. Interv. Sch. Clin..

[7] Yuzaidey N.A.M., Din N.C., Ahmad M., Ibrahim N., Razak R.A., Harun D. (2018). Interventions for children with dyslexia: A review on current intervention methods. Med. J. Malays..

[8] Ortiz A., Martinez-Murcia F.J., Luque J.L., Giménez A., Morales-Ortega R., Ortega J. (2020). Dyslexia Diagnosis by EEG Temporal and Spectral Descriptors: An Anomaly Detection Approach. Int. J. Neural Syst..

[9] Fletcher J.M., Francis D.J., Foorman B.R., Schatschneider C. (2021). Early Detection of Dyslexia Risk: Development of Brief, Teacher-Administered Screens. Learn. Disabil. Q..

[10] Tosun D., Arikan S., Babür N. (2021). Teachers’ Knowledge and Perception about Dyslexia: Developing and Validating a Scale. Int. J. Assess. Tools Educ..

[11] Chakraborty S., Dasgupta A., Routray A. (2020). Localization of eye Saccadic signatures in Electrooculograms using sparse representations with data driven dictionaries. Pattern Recognit. Lett..

[12] Mulam H., Mudigonda M. (2020). EOG-based eye movement recognition using GWO-NN optimization. Biomed. Tech..

[13] Kumar D., Sharma A. Electrooculogram-based virtual reality game control using blink detection and gaze calibration. Proceedings of the 2016 International Conference on Advances in Computing, Communications and Informatics, ICACCI 2016.

[14] Fatourechi M., Bashashati A., Ward R.K., Birch G.E. (2007). EMG and EOG artifacts in brain computer interface systems: A survey. Clin. Neurophysiol..

[15] Usakli A.B., Gurkan S., Aloise F., Vecchiato G., Babiloni F. (2010). On the use of electrooculogram for efficient human computer interfaces. Comput. Intell. Neurosci..

[16] Usakli A.B., Gurkan S. (2010). Design of a novel efficient humancomputer interface: An electrooculagram based virtual keyboard. IEEE Trans. Instrum. Meas..

[17] Lee K.R., Chang W.D., Kim S., Im C.H. (2017). Real-time eye-writing recognition using electrooculogram. IEEE Trans. Neural Syst. Rehabil. Eng..

[18] Latifoǧlu F., Esas M.Y., Demirci E. (2020). Diagnosis of attention-deficit hyperactivity disorder using EOG signals: A new approach. Biomed. Tech..

[19] Altıntop Ç.G., Latifoğlu F., Akın A.K. (2022). Can patients in deep coma hear us? Examination of coma depth using physiological signals. Biomed. Signal Process. Control.

[20] Eden G.F., Stein J.F., Wood H.M., Wood F.B. (1994). Differences in eye movements and reading problems in dyslexic and normal children. Vis. Res..

[21] Martos F.J., Vila J. (1990). Differences in eye movements control among dyslexic, retarded and normal readers in the Spanish population. Read. Writ..

[22] Bachmann C., Mengheri L. (2018). Dyslexia and fonts: Is a specific font useful?. Brain Sci..

[23] Rello L., Ballesteros M. Detecting readers with dyslexia using machine learning with eye tracking measures. Proceedings of the W4A 2015—12th Web for All Conference.

[24] Rello L., Baeza-Yates R. (2016). The effect of font type on screen readability by people with dyslexia. ACM Trans. Access. Comput..

[25] Felicia G. (2024). December the Science Behind Dyslexia Fonts and Their Effectiveness. https://dyslexichelp.org/why-does-dyslexia-font-work/.

[26] Kuster S.M., van Weerdenburg M., Gompel M., Bosman A.M.T. (2018). Dyslexie font does not benefit reading in children with or without dyslexia. Ann. Dyslexia.

[27] Aksan D. (1995). Her Yönüyle Dil: Ana Çizgileriyle Dilbilim.

[28] Kargin T., Güldenoğlu B., Sümer H.M. (2019). Morfolojik Farkındalık Becerilerinin Okuma Sürecindeki Rolünün Gelişimsel Bakış Açısıyla İncelenmesi: İşiten ve İşitme Engelli Okuyuculardan Bulgular. Ank. Üniversitesi Eğitim Bilim. Fakültesi Özel Eğitim Derg..

[29] Güven S., Friedmann N. (2019). Developmental Letter Position Dyslexia in Turkish, a Morphologically Rich and Orthographically Transparent Language. Front. Psychol..

[30] Acartürk C., Özkan A., Pekçetin T.N., Ormanoğlu Z., Kırkıcı B. (2024). TURead: An eye movement dataset of Turkish reading. Behav. Res. Methods.

[31] Güven S., Friedmann N. (2021). Vowel dyslexia in Turkish: A window to the complex structure of the sublexical route. PLoS ONE.

[32] Sümer Dodur H.M., Altindağ Kumaş Ö. (2021). Knowledge and beliefs of classroom teachers about dyslexia: The case of teachers in Turkey. Eur. J. Spec. Needs Educ..

[33] Yavuz T., Yavuz I.S., Deveci B., Fidan T. (2022). Dyslexia in education in Turkey. The Routledge International Handbook of Dyslexia in Education.

[34] Schulte-Körne G. (2010). The Prevention, Diagnosis, and Treatment of Dyslexia. Dtsch. Ärzteblatt Int..

[35] Shaywitz S.E., Shaywitz B.A. (2005). Dyslexia (specific reading disability). Biol. Psychiatry.

[36] Usman O.L., Muniyandi R.C., Omar K., Mohamad M. (2021). Advance Machine Learning Methods for Dyslexia Biomarker Detection: A Review of Implementation Details and Challenges. IEEE Access.

[37] Zahia S., Garcia-Zapirain B., Saralegui I., Fernandez-Ruanova B. (2020). Dyslexia detection using 3D convolutional neural networks and functional magnetic resonance imaging. Comput. Methods Programs Biomed..

[38] Ileri R., Latifoğlu F., Demirci E. (2022). A novel approach for detection of dyslexia using convolutional neural network with EOG signals. Med. Biol. Eng. Comput..

[39] Rello L., Romero E., Ali A., Williams K., Bigham J.P., White N.C. (2018). Screening dyslexia for english using HCI measures and machine learning. ACM International Conference Proceeding Series.

[40] Spoon K., Siek K., Crandall D., Fillmore M. Can We (and Should We) Use AI to Detect Dyslexia in Children’s Handwriting?. Proceedings of the International Conference on Machine Learning AI for Social Good Workshop (NeurIPS 2019).

[41] Benfatto M.N., Seimyr G.Ö., Ygge J., Pansell T., Rydberg A., Jacobson C. (2016). Screening for dyslexia using eye tracking during reading. PLoS ONE.

[42] Nerušil B., Polec J., Škunda J., Kačur J. (2021). Eye tracking based dyslexia detection using a holistic approach. Sci. Rep..

[43] Rayner K., Fischer M.H. (1996). Mindless reading revisited: Eye movements during reading and scanning are different. Percept. Psychophys..

[44] Schmeisser E.T., Mcdonough J.M., Bond M., Hislop P.D., Epstein A.D. (2001). Fractal analysis of eye movements during reading. Optom. Vis. Sci..

[45] Biscaldi M., Fischer B., Aiple F. (1994). Saccadic eye movements of dyslexic and normal reading children. Perception.

[46] Latifoğlu F., İleri R., Demirci E. (2021). Assessment of dyslexic children with EOG signals: Determining retrieving words/re-reading and skipping lines using convolutional neural networks. Chaos Solitons Fractals.

[47] Sammaiah A., Narsimha B., Suresh E., Sanjeeva Reddy M. On the performance of wavelet transform improving Eye blink detections for BCI. Proceedings of the 2011 International Conference on Emerging Trends in Electrical and Computer Technology, ICETECT 2011.

[48] Schleicher R., Galley N., Briest S., Galley L. (2008). Blinks and saccades as indicators of fatigue in sleepiness warnings: Looking tired?. Ergonomics.

[49] Kong X., Wilson G.F. (1998). A new EOG-based eyeblink detection algorithm. Behav. Res. Methods Instrum. Comput..

[50] Venkataramanan S., Prabhat P., Choudhury S.R., Nemade H.B., Sahambi J. Biomedical instrumentation based on Electrooculogram (EOG) signal processing and application to a hospital alarm system. Proceedings of the Proceedings—2005 International Conference on Intelligent Sensing and Information Processing, ICISIP’05.

[51] Sinha S., Routh P.S., Anno P.D., Castagna J.P. (2005). Spectral decomposition of seismic data with continuous-wavelet transform. Geophysics.

[52] Darvishi S., Al-Ani A. Brain-computer interface analysis using continuous wavelet transform and adaptive neuro-fuzzy classifier. Proceedings of the Annual International Conference of the IEEE Engineering in Medicine and Biology—Proceedings.

[53] Türk Ö., Özerdem M.S. (2019). Epilepsy detection by using scalogram based convolutional neural network from eeg signals. Brain Sci..

[54] Leao R.N., Burne J.A. (2004). Continuous wavelet transform in the evaluation of stretch reflex responses from surface EMG. J. Neurosci. Methods.

[55] Wang T., Lu C., Sun Y., Yang M., Liu C., Ou C. (2021). Automatic ECG classification using continuous wavelet transform and convolutional neural network. Entropy.

[56] Elhassouny A., Smarandache F. The History Began from AlexNet A. Proceedings of the International Conference of Computer Science and Renewable Energies (ICCSRE).

[57] Shafiq M., Gu Z. (2022). Deep Residual Learning for Image Recognition: A Survey. Appl. Sci..

[58] Howard A.G., Zhu M., Chen B., Kalenichenko D., Wang W., Weyand T., Andreetto M., Adam H. (2017). MobileNets. arXiv.

[59] Shaywitz B.A., Lyon G.R., Shaywitz S.E. (2006). The role of functional magnetic resonance imaging in understanding reading and dyslexia. Dev. Neuropsychol..

[60] Christodoulides P., Miltiadous A., Tzimourta K.D., Peschos D., Ntritsos G., Zakopoulou V., Giannakeas N., Astrakas L.G., Tsipouras M.G., Tsamis K.I. (2022). Classification of EEG signals from young adults with dyslexia combining a Brain Computer Interface device and an Interactive Linguistic Software Tool. Biomed. Signal Process. Control.

[61] Rauschenberger M., Rello L., Baeza-Yates R., Bigham J.P. Towards language independent detection of dyslexia with a web-based game. Proceedings of the 15th Web for All Conference: Internet of Accessible Things 2018, W4A 2018.

[62] Rauschenberger M., Baeza-Yates R., Rello L. (2022). A Universal Screening Tool for Dyslexia by a Web-Game and Machine Learning. Front. Comput. Sci..

[63] Ullah Khan R., Lee J., Cheng A., Bee O.Y. (2018). Machine Learning and Dyslexia: Diagnostic and Classification System (DCS) for Kids with Learning Disabilities. Int. J. Eng. Technol..

[64] Jothi Prabha A., Bhargavi R. (2022). Prediction of Dyslexia from Eye Movements Using Machine Learning. IETE J. Res..

[65] Abdul Hamid S.S., Admodisastro N., Manshor N., Kamaruddin A., Ghani A.A.A. (2018). Dyslexia adaptive learning model: Student engagement prediction using machine learning approach. Advances in Intelligent Systems and Computing.

[66] El Hmimdi A.E., Ward L.M., Palpanas T., Kapoula Z. (2021). Predicting dyslexia and reading speed in adolescents from eye movements in reading and non-reading tasks: A machine learning approach. Brain Sci..

[67] Aldehim G., Rashid M., Alluhaidan A.S., Sakri S., Basheer S. (2024). Deep Learning for Dyslexia Detection: A Comprehensive CNN Approach with Handwriting Analysis and Benchmark Comparisons. J. Disabil. Res..

[68] Spoon K., Crandall D., Siek K. Towards Detecting Dyslexia in Children’s Handwriting Using Neural Networks. Proceedings of the International Conference on Machine Learning AI for Social Good Workshop.

[69] Taş T., Bülbül M.A., HaşimOğlu A., Meral Y., Çalişkan Y., Budagova G., Kutlu M. (2023). A machine learning approach for dyslexia detection using Turkish audio records. Turk. J. Electr. Eng. Comput. Sci..

[70] Latifoglu F., Ileri R., Demirci E., Altintop C.G. Detection of Reading Movement from EOG Signals. Proceedings of the 2020 IEEE International Symposium on Medical Measurements and Applications (MeMeA).

[71] Ileri R., Latifoglu F., Demirci E. New Method to Diagnosis of Dyslexia Using 1D-CNN. Proceedings of the TIPTEKNO 2020—Tip Teknolojileri Kongresi—2020 Medical Technologies Congress, TIPTEKNO 2020.

