# Supplementary material for: DyslexiaNet: Examining the Viability and Efficacy of Eye Movement-Based Deep Learning for Dyslexia Detection
Source: J Eye Mov Res. 2025 Oct 15;18(5):56. doi: 10.3390/jemr18050056 (PMC12565239; doi:10.3390/jemr18050056)
Supplement: Supplementary file 1 [file jemr-18-00056-s001.zip › jemr-3780297-supplementary.pdf]

Supporting Information

DyslexiaNet: Examining the Viability and Efficacy of Eye Movement-Based Deep Learning for Dyslexia Detection

Ramis İleri[1],

ramissileri@gmail.com

Çiğdem Gülüzar Altıntop[1,*],

cigdemacer@erciyes.edu.tr

Fatma Latifoğlu[1,*],

flatifoglu@erciyes.edu.tr

Esra Demirci[3]

esrademirci@erciyes.edu.tr

[1]Department of Biomedical Engineering, Faculty of Engineering, Erciyes University, TURKIYE

[3]Department of Child and Adolescent Psychiatry, Erciyes University School of Medicine, TURKIYE

Supplementary Table S1. Detailed information about the texts

	Number of lines	Number of minimum words	Number of maximum words	Number of average words
**2nd grade**	4	23	25	23,5
**3rd grade**	5	28	30	30,06
**4th grade**	5	30	32	31,31

Text No	2nd Grade		3rd Grade		4th Grade	
	Font Size	Typeface	Font Size	Typeface	Font Size	Typeface
1	18	Kelvinch	16	Kelvinch	14	Kelvinch
2	20	Kelvinch	18	Kelvinch	16	Kelvinch
3	22	Kelvinch	20	Kelvinch	18	Kelvinch
4	24	Kelvinch	22	Kelvinch	20	Kelvinch
5	18	TTKB Dik Temel Abece	16	Arial	14	Arial
6	20	TTKB Dik Temel Abece	18	Arial	16	Arial
7	22	TTKB Dik Temel Abece	20	Arial	18	Arial
8	24	TTKB Dik Temel Abece	22	Arial	20	Arial
9	18	Arial	16	Times New Roman	14	Times New Roman
10	20	Arial	18	Times New Roman	16	Times New Roman
11	22	Arial	20	Times New Roman	18	Times New Roman

12	24	Arial	22	Times New Roman	20	Times New Roman
13	18	Times New Roman	16	BonvecoCF	14	BonvecoCF
14	20	Times New Roman	18	BonvecoCF	16	BonvecoCF
15	22	Times New Roman	20	BonvecoCF	18	BonvecoCF
16	24	Times New Roman	22	BonvecoCF	20	BonvecoCF
17	18	BonvecoCF	16	SofiaPro	14	SofiaPro
18	20	BonvecoCF	18	SofiaPro	16	SofiaPro
19	22	BonvecoCF	20	SofiaPro	18	SofiaPro
20	24	BonvecoCF	22	SofiaPro	20	SofiaPro
21	20	TTKB Dik Temel Abece (Colored)	16	TTKB Dik Temel Abece	14	TTKB Dik Temel Abece
22	20	Kelvinch(Colored)	18	TTKB Dik Temel Abece	16	TTKB Dik Temel Abece
23	20	Arial (Colored)	20	TTKB Dik Temel Abece	18	TTKB Dik Temel Abece
24	20	BonvecoCF (Colored)	22	TTKB Dik Temel Abece	20	TTKB Dik Temel Abece
25	20	Times New Roman (Colored)	20	TTKB Dik Temel Abece(Colored)	20	TTKB Dik Temel Abece (Colored)
26	20	Times New Roman (Bold)	20	Kelvinch(Colored)	20	Kelvinch (Colored)
27	20	Times New Roman (İtalic)	20	Times New Roman (Colored)	20	Times New Roman (Colored)
28	20	TTKB Dik Temel Abece (Colored)	20	Arial (Colored)	20	Arial (Colored)
29			20	BonvecoCF (Colored)	20	BonvecoCF(Colored)
30			20	Times New Roman (Bold)	20	Times New Roman (Bold)
31			20	Times New Roman (İtalic)	20	Times New Roman (İtalic)
32			20	Times New Roman (line pitch=1)	20	Times New Roman (line pitch=1)

Supplementary Table S2. Subject's characteristics

	Dyslexia	Healthy	p
Sample size	23	13	
Age(years)	8,9565	8,5385	0,188ns
	(8-11)	(8-11)	
Gender	13/10	7/6	0,881ns
(Female/Male)			

Groups were compared with independent sample t tests. Abbreviations: p, p value; ns, non-significance.

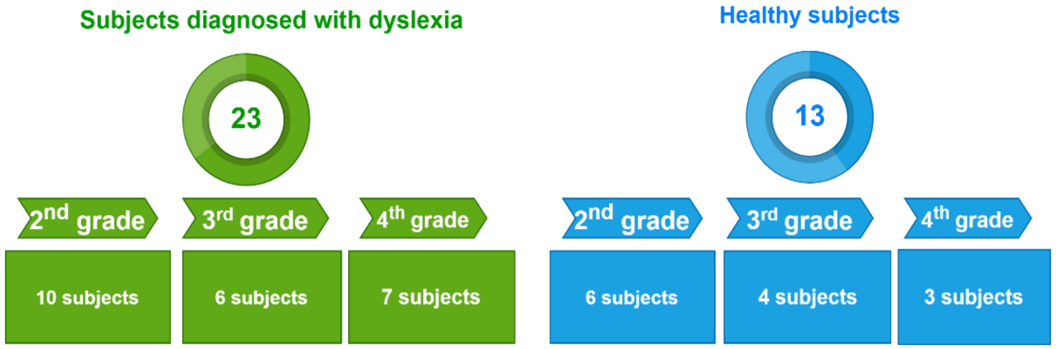

Supplementary Figure S1. A summary of the subjects according to their health status

Supplementary Table S3 Hyperparameter of the DyslexiaNet

No.	Parameters	Values
1	Optimizer	Adam
2	Learning rate	0.001
3	MiniBatch size	32
4	Epoch	2

Supplementary Table S4 Definition of two different channels

Channel	Data	Input Size
Channel 1	Vertical EOG Scalogram Image	6000 (Healthy class=3000; Dyslexia class=3000)
Channel 2	Horizontal EOG Scalogram Image	6000 (Healthy class=3000; Dyslexia class=3000)

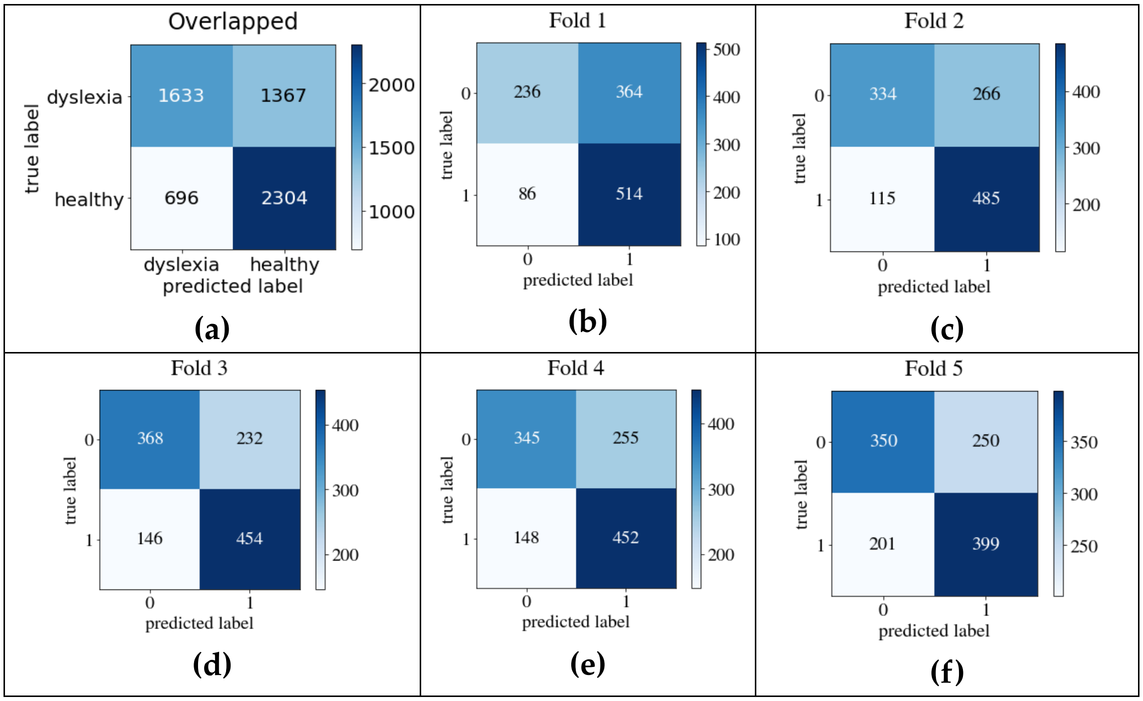

Supplementary Figure S2. Channel 1 overlapping and 5-fold confusion matrices for *AlexNet*: (a) overlapped CM, (b) Fold 1 CM, (c) Fold 2 CM, (d) Fold 3 CM, (d) Fold 4 CM, (f) Fold 5 CM

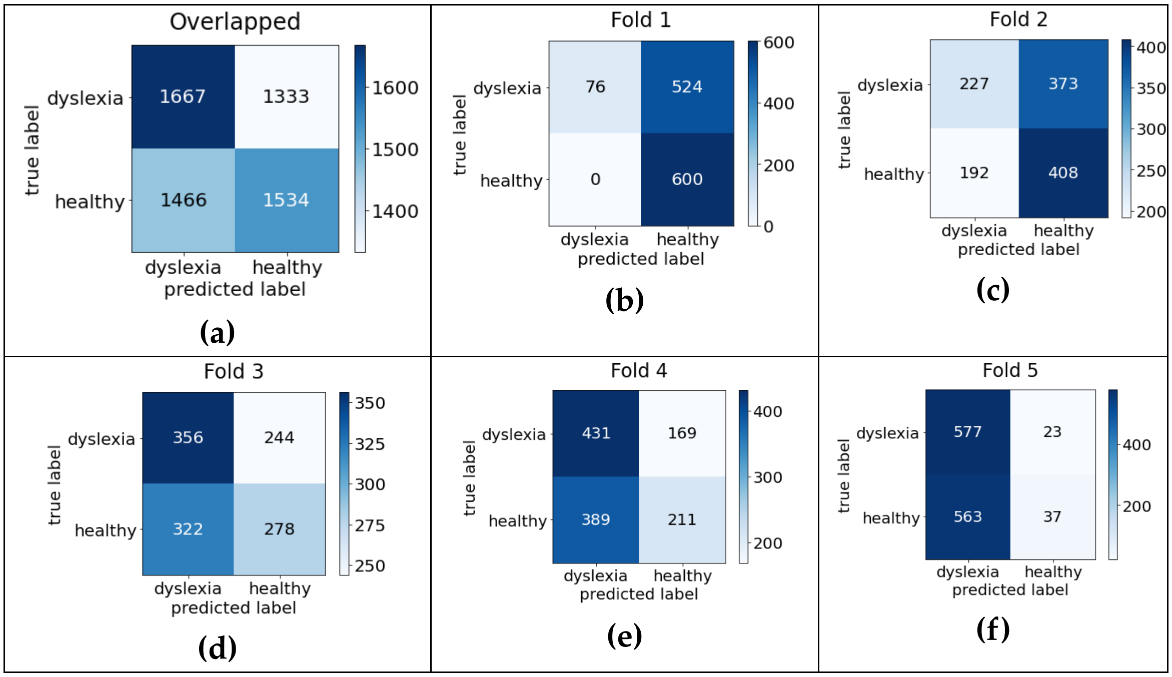

Supplementary Figure S3. Channel 1 overlapping and 5-fold confusion matrices for *ResNet50*: (a) overlapped CM, (b) Fold 1 CM, (c) Fold 2 CM, (d) Fold 3 CM, (d) Fold 4 CM, (f) Fold 5 CM

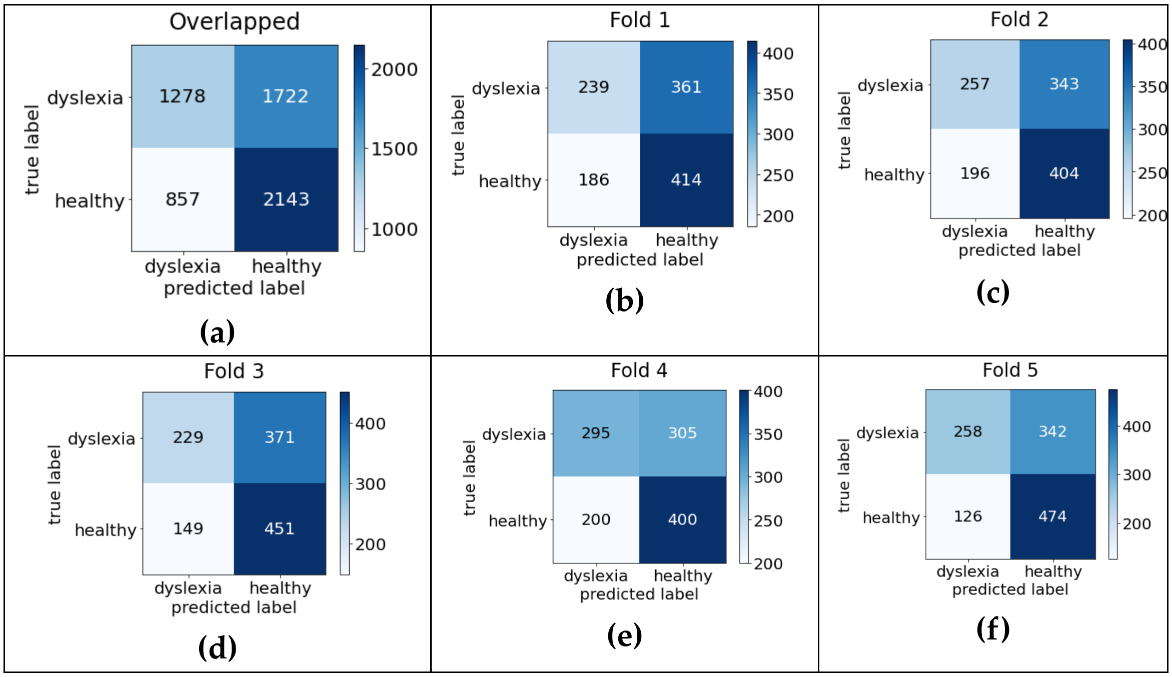

Supplementary Figure S4. Channel 1 overlapping and 5-fold confusion matrices for

MobileNet: (a) overlapped CM, (b) Fold 1 CM, (c) Fold 2 CM, (d) Fold 3 CM, (d) Fold 4 CM, (f) Fold 5 CM

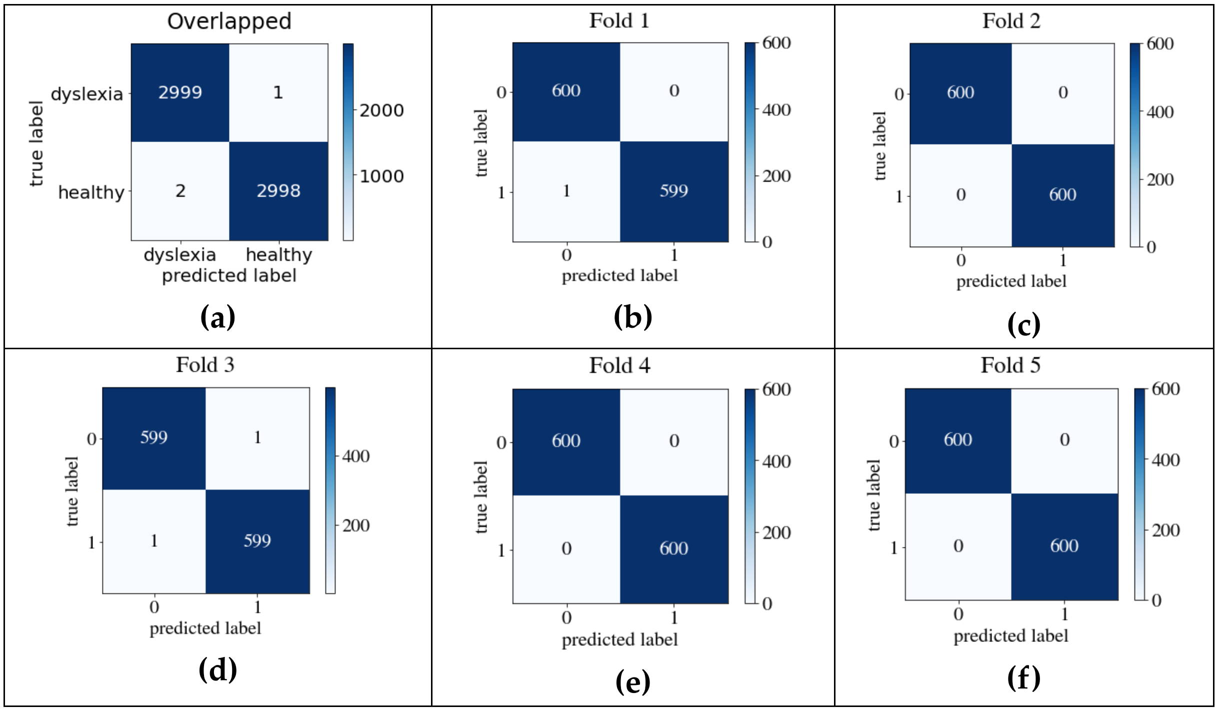

Supplementary Figure S5. Channel 2 overlapping and 5-fold confusion matrices for *AlexNet*: (a) overlapped CM, (b) Fold 1 CM, (c) Fold 2 CM, (d) Fold 3 CM, (d) Fold 4 CM, (f) Fold 5 CM

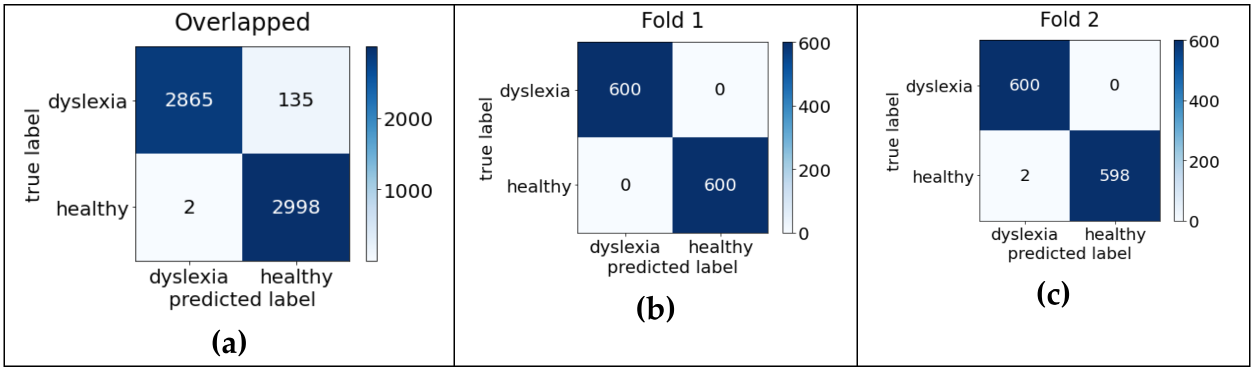

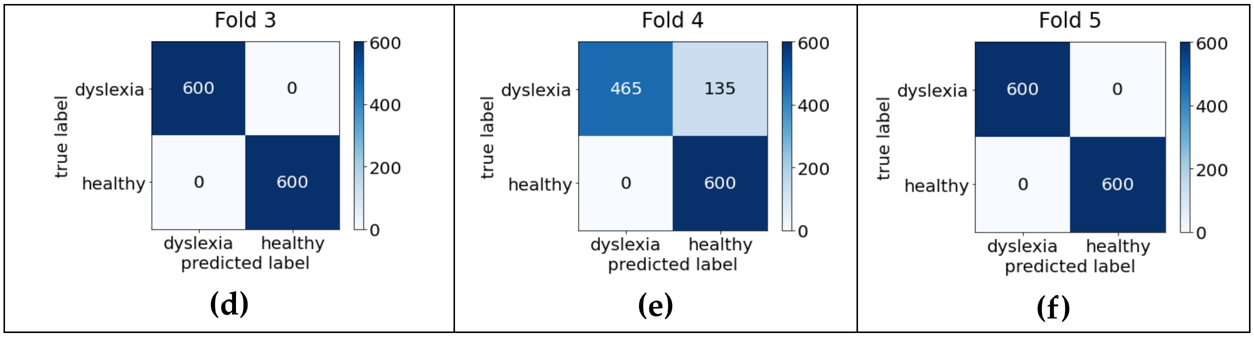

Supplementary Figure S6. Channel 2 overlapping and 5-fold confusion matrices for *ResNet50*: (a) overlapped CM, (b) Fold 1 CM, (c) Fold 2 CM, (d) Fold 3 CM, (d) Fold 4 CM, (f) Fold 5 CM

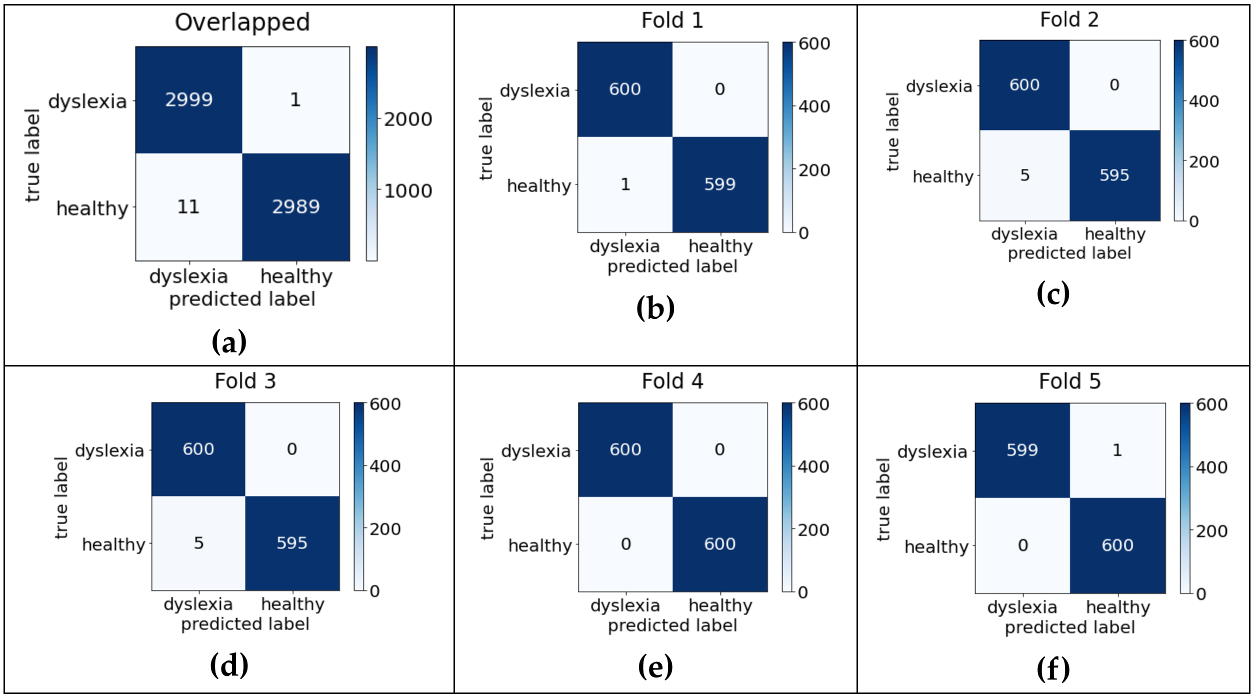

Supplementary Figure S7. Channel 2 overlapping and 5-fold confusion matrices for *MobileNet*: (a) overlapped CM, (b) Fold 1 CM, (c) Fold 2 CM, (d) Fold 3 CM, (d) Fold 4 CM, (f) Fold 5 CM

Supplementary Table S5. The fundamental parameters of networks

Parameter	ResNet-50	AlexNet	MobileNetV2	DyslexiaNet
Number of Layers	50	8	53	5

Connected Layers	1 fully connected layer	3 fully connected layers	1 fully connected layer	1 fully connected layer
Activation Function	ReLU	ReLU	ReLU or PReLU	ReLU
Optimizer	Adam	Adam	Adam	Adam
Dropout	0.5	0.5	0.5	0.5
Batch Size	128	128	128	32
Loss Function	Cross-Entropy	Cross-Entropy	Cross-Entropy	Cross-Entropy
